# Designing Bioorthogonal Reactions for Biomedical Applications

**DOI:** 10.34133/research.0251

**Published:** 2023-12-15

**Authors:** Qingfei Zhang, Gaizhen Kuang, Li Wang, Ping Duan, Weijian Sun, Fangfu Ye

**Affiliations:** ^1^ Wenzhou Institute, University of Chinese Academy of Sciences, Wenzhou 325001, China.; ^2^ Beijing National Laboratory for Condensed Matter Physics, Institute of Physics, Chinese Academy of Sciences, Beijing 100190, China.; ^3^ Department of Obstetrics and Gynecology, The Second Affiliated Hospital and Yuying Children’s Hospital of Wenzhou Medical University, Wenzhou, Zhejiang 325027, China.; ^4^ Department of Gastrointestinal Surgery, The Second Affiliated Hospital and Yuying Children’s Hospital of Wenzhou Medical University, Wenzhou, 325027, China.

## Abstract

Bioorthogonal reactions are a class of chemical reactions that can be carried out in living organisms without interfering with other reactions, possessing high yield, high selectivity, and high efficiency. Since the first proposal of the conception by Professor Carolyn Bertozzi in 2003, bioorthogonal chemistry has attracted great attention and has been quickly developed. As an important chemical biology tool, bioorthogonal reactions have been applied broadly in biomedicine, including bio-labeling, nucleic acid functionalization, drug discovery, drug activation, synthesis of antibody–drug conjugates, and proteolysis-targeting chimeras. Given this, we summarized the basic knowledge, development history, research status, and prospects of bioorthogonal reactions and their biomedical applications. The main purpose of this paper is to furnish an overview of the intriguing bioorthogonal reactions in a variety of biomedical applications and to provide guidance for the design of novel reactions to enrich bioorthogonal chemistry toolkits.

## Introduction

**Fig. 1.**
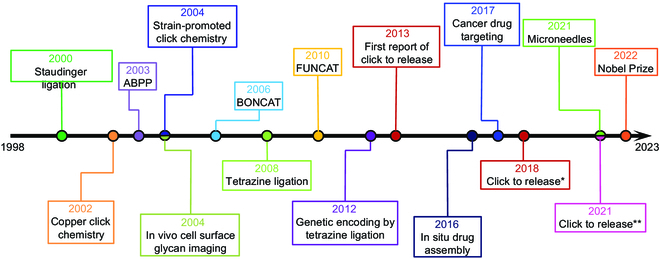
Bioorthogonal chemistry’s path to the Nobel Prize. Reproduced with permission [9]. Copyright 2022, American Chemical Society.

Bioorthogonal reactions are chemical reactions that can take place inside living organisms without interfering with native biological processes [1,2]. These reactions utilize the principles of click chemistry, which is a modular approach to chemical synthesis that involves highly selective and efficient reactions [3,4]. K. Barry Sharpless first coined the term “click chemistry”, which refers to a class of reactions that entail the formation of carbon-heteroatomic bonds (C-X-C) [5,6]. Subsequently, Carolyn Bertozzi took click chemistry to a new dimension and developed the concept of bioorthogonal chemistry [7]. Orthogonal means non-interference, implying a chemical reaction can proceed independently in the biological context (e.g., living cells and living animals) without affecting the surrounding biological system, and the various substances in the biological system will not interfere with it. In general, the pair of reactants involved in bioorthogonal reactions recognize each other and ignore the other molecules around them, maintaining a high degree of specificity [8]. Therefore, the bioorthogonal reactions possess the superiorities of high yield, high selectivity, high efficiency, and no side reactions in biological environments [9]. Over the past 2 decades, bioorthogonal chemistry has experienced substantial advancements, resulting in its widespread application. Figure 1 presents a comprehensive timeline highlighting the remarkable advancements and applications of bioorthogonal chemistry. The Nobel Prize in Chemistry for 2022 recognized the remarkable contributions of American scientists Carolyn Bertozzi and Karl Barry Sharpless, as well as Danish scientist Morten Meldal, to the development of click chemistry and bioorthogonal chemistry [10,11].

**Fig. 2.**
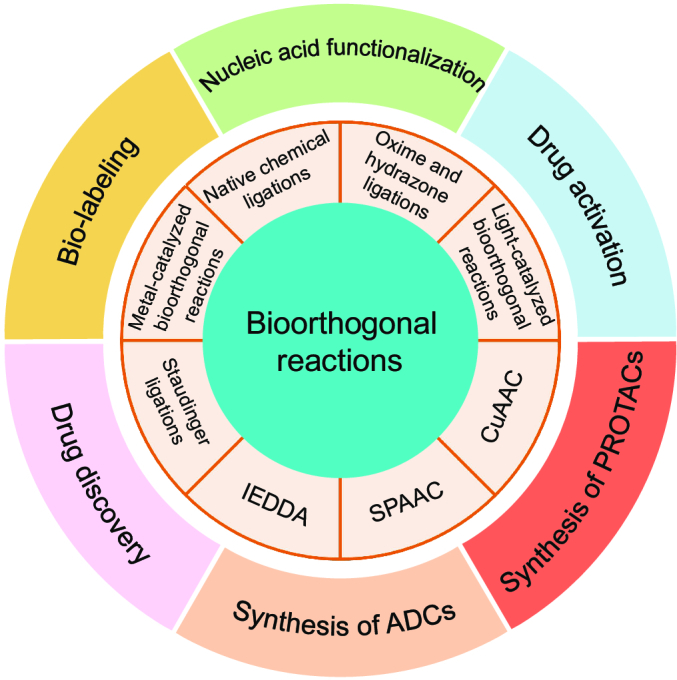
Schematic diagram of bioorthogonal reactions and their biomedical applications.

Bioorthogonal chemistry can probe biological systems through selective covalent bond formation with minimal interference to the system under study [12,13]. With the development of chemical technology, many kinds of bioorthogonal reactions have been discovered and successfully applied in diverse biomedical fields. The classical bioorthogonal reactions include native chemical ligations [14,15], oxime/hydrazone ligations [16,17], Staudinger ligations [18,19], Cu(I) catalyzed azide–alkyne cycloadditions (CuAAC) [20,21], strain-promoted azide–alkyne cycloadditions (SPAAC) [22], inverse electron-demand Diels–Alder reactions (IEDDA) [23], light-catalyzed bioorthogonal reactions [24], metal-catalyzed bioorthogonal reactions [25], etc., as illustrated in Fig. 2. These reactions have found extensive application in cellular systems and living animals, showing unparalleled advantages in biomedical imaging, protein synthesis, pharmaceutical chemistry, materials science, polymer science, etc. [2,9,26]. Besides, they are extensively employed in genetic codon amplification techniques, metabolic engineering, antibody-conjugated drugs, drug target recognition, proteolysis-targeting chimera (PROTAC), and drug delivery systems (Fig. 2) [7]. In this review, we first introduce various reactions that are included in the toolbox of bioorthogonal chemistry. Then, we highlight the wide range of applications for which these chemical reactions can be utilized. Finally, the current limitations of bioorthogonal chemistry are discussed and we provide an outlook for this exciting research area and its development in the next decade.

## Various Bioorthogonal Reactions

### Bioorthogonal reactions without catalysis

#### Native chemical ligations

**Fig. 3.**
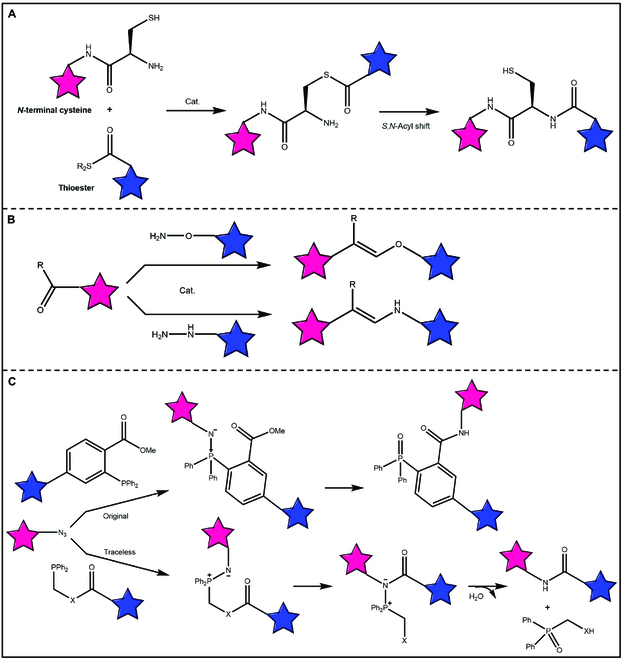
(A) The native chemical ligation. Native chemical ligation is achieved by a catalytic reaction between a thioester and an amino terminal cysteine residue, with *S,N*-acyl group translocation through a thioester intermediate to form a natural amide bond. (B) The oxime and hydrazine ligations. Using aniline-based catalysts, the connection between oxime and hydrazone occurs between the carbonyl group and hydroxylamine or hydrazine. (C) The Staudinger ligation. Both original and traceless Staudinger ligation can form natural amide bonds between an azide group and a carbonyl group.

Natural chemical ligation refers to a reaction wherein peptide 1, featuring a thioester at the C-terminal, is coupled to peptide 2 containing a cysteine residue at the N-terminal (Fig. 3A) [27,28]. Under the action of the catalyst, the mercapto group of peptide 2 attacks the thioester in peptide 1, and a transesterification reaction occurs at room temperature to obtain new thioester intermediates. Subsequently, the intermediate undergoes an intramolecular *S,N*-acyl transfer reaction, resulting in the formation of a native peptide bond. In chemical ligating, nucleophilic catalysts like thiols or imidazole can be employed to accelerate the rate of the final transesterification step [29]. After ligature, the internal cysteine can be converted to alanine through the desulfurization reaction [30]. The use of selenocysteine and selenopeptide can accelerate the rate of these linking reactions, and selenocysteine can serve as a substitute for alanine in complex target synthesis due to its ability to undergo deselenization in the presence of unprotected cysteine and methionine residues [31]. Expressed protein ligation represents an alternative form of natural chemical ligation, involving the fusion of a segment containing a C-terminal thioester with an intein through a recombinant protein [32]. These methods provide a strategy to synthesize proteins with precise site-specific post-translational modifications and incorporate unnatural amino acids, including the synthesis and semi-synthesis of proteins that surpass the limitations of automated peptide synthesis due to their size.

Several methods, such as serine–threonine ligation, which involves a C-terminal *o-*formyl phenolate interacting with an N-terminal serine or threonine, have been devised as complementary approaches to native chemical ligation [33,34]. Besides, the functional interaction between the C-terminal ketyl-hydroxylamine and the N-terminal hydroxylamine is a component of the *o*-ketyl-hydroxylamine connection, as is the potassium acyltrifluoroborate (KAT) connection between *o-*carbamoyl hydroxylamine and potassium trifluoroylborate [35]. In addition, the specific transformation of the N-terminus in a protein can be achieved through straight convergence of N-terminal cysteine and serine, or tryptophan side-chain nucleophiles with aldehydes to produce cyclical compounds [36,37]. In conversion interactions using Rapoport’s salt or pyridoxal-5′-phosphate, the N-terminal is transformed into an aldehyde or ketone, which subsequently can create an oxime [38,39]. Additionally, for the production of the cyclical imidazolidinone products, 2-pyridine carboxaldehyde analogs can also be utilized in one-step N-terminal linkage [40]. Native chemical ligation techniques, along with their variants for the formation of native amide bonds, have extensive applications in the synthesis and semi-synthesis of peptides and proteins, particularly when integrated with solid-phase peptide synthesis [41].

#### Oxime/hydrazone ligations

The ligation reactions by forming oxime and hydrazone are regarded as some of the efficient bioorthogonal reactions for biological ligation applications. Oximes and hydrazones are formed through the condensation of carbonyl groups with alkoxy amines and hydrazines, respectively (Fig. 3B) [16,42]. The obtained compounds exhibit greater stability toward hydrolysis than imines due to the presence of heteroatoms attached to *sp2* hybrid nitrogen atoms [43]. However, these compounds can undergo reversible hydrolysis under physiological conditions, particularly under weakly acidic conditions (pH 5 to 7), leading to their conversion into aldehydes/ketones and alkoxy amines/hydrazine [44]. Therefore, according to this property, the coupled fragments can be released by regulating the pH of the environment in which the conjugate is located. Oxime/hydrazone ligations are currently used in the extracellular environment, such as cell surface labeling, and in the synthesis of some antibody-coupled drugs [45–47].

#### Staudinger ligations

Staudinger ligation reactions were first discovered in 1919. The researchers found that azide and triphenylphosphine can be converted into amine and triphenylphosphine in an aqueous environment (Fig. 3C) [18,48]. In 2000, Bertozzi et al. developed a novel bioorthogonal reaction based on the principle of this reaction, in which triphenylphosphine derivatives labeled with orthoformates and 2 macromolecular fragments labeled with azide functional groups were coupled together through Staudinger ligation, and the target ligation product was obtained through intramolecular rearrangement [49]. This method is the first reported bioorthogonal reaction for biotinizing polysaccharides on the surface of cell membranes. Subsequently, Bertozzi developed a “traceless” Staudinger reaction [50], in which a diphenylphosphine derivative labeled with an ester/amide/thioester and 2 macromolecular fragments labeled with an azide functional group were coupled together, followed by intramolecular rearrangement and further hydrolysis to form a conjugate connected only by an amide bond. This reaction was subsequently applied to the modification of nucleic acids, proteins, and other biomolecules [51–54].

#### Strain-promoted azide–alkyne cycloadditions

**Fig. 4.**
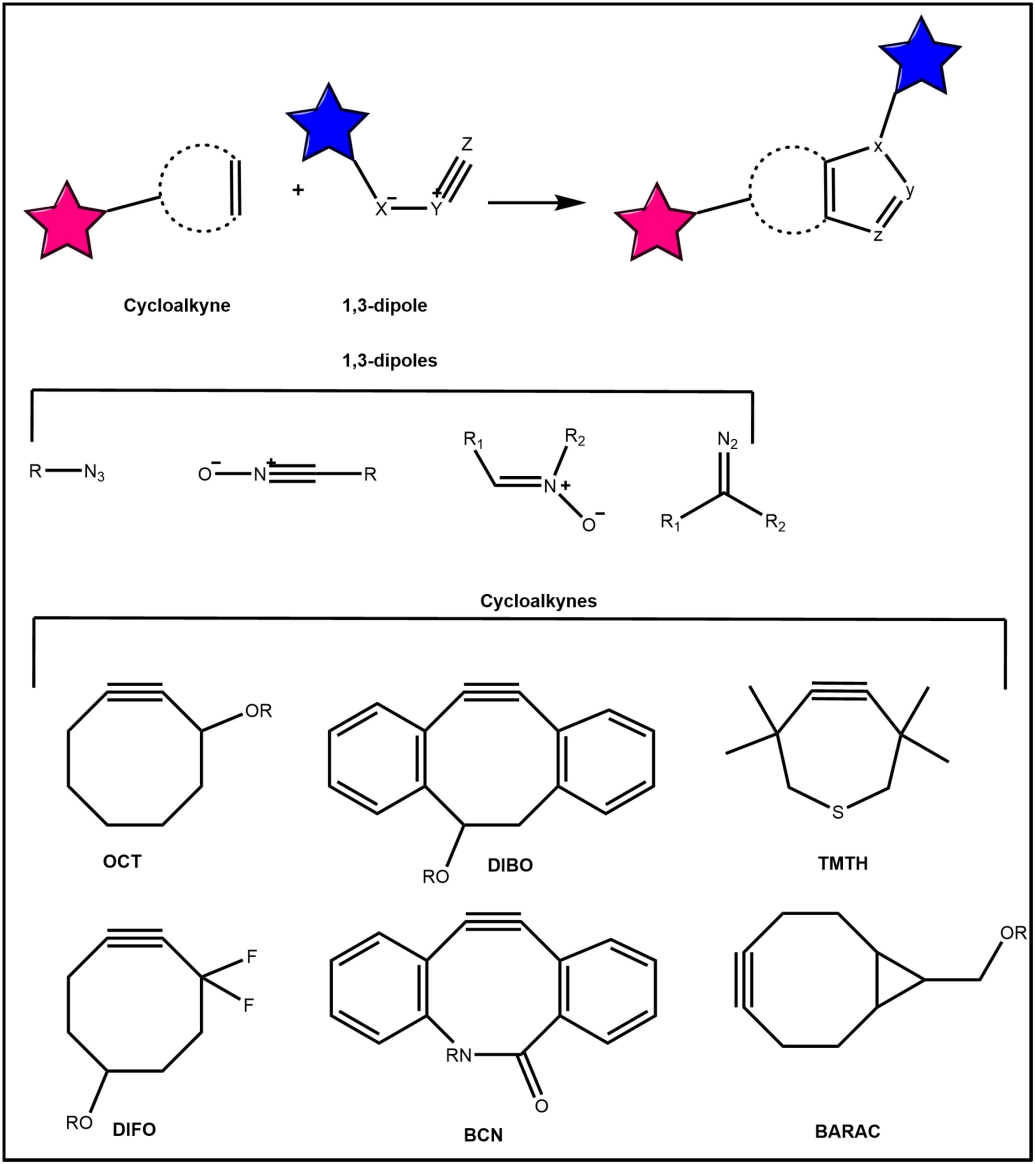
The SPAAC. The strained alkyne and dipole could achieve copper-free catalyzed cycloaddition. OCT, cyclooctyne; DIBO, dibenzocyclooctyne; TMTH, 3,3,6,6-tetramethylthiaheptyne; DIFO, difluorinated cyclooctyne; BCN, bicyclo[2.1.0]nonyne; BARAC, biarylazacyclooctynone.

In the CuAAC reaction, the used Cu(I) will react with the sulfhydryl group in the organism, resulting in biological toxicity. Consequently, its application is limited to in vitro experiments. To address this challenge, Bertozzi and colleagues employed high-tension cyclooctyne derivatives as the substrates for the reaction. They used the ring tension released when the triple bond of cyclooctyne became a double bond to achieve an efficient reaction without the addition of a copper catalyst (Fig. 4) [55]. Therefore, Bertozzi gave this reaction a famous name, i.e., SPAAC. In addition, apart from azide, 1,3-dipoles can also select bicyclic derivatives, benzannulated cyclooctynes, and benzannulated cyclooctynes [56,57]. Since there is no external catalyst and small groups are introduced, these artificially introduced click chemical reactions do not affect other biochemical reactions in normal physiological processes, and are very suitable for the labeling of targets in the complex environment of organisms [12]. Thus, Professor Bertozzi drew on the concept of “orthogonal” in mathematics and proposed bioorthogonal chemistry based on click chemistry, which greatly expanded the application potential of click chemistry in the area of biomedicine.

#### Inverse electron-demand Diels–Alder reactions

**Fig. 5.**
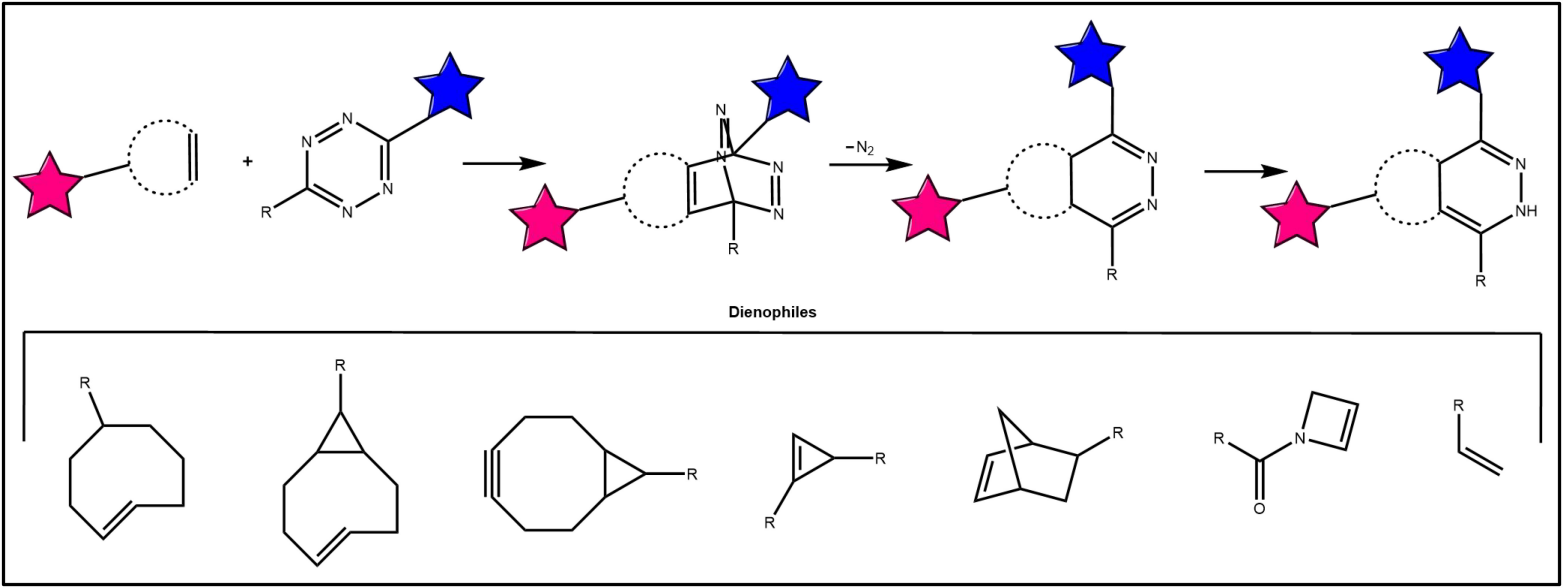
The IEDDA. The inverse-electron demand [4+2] cycloadditions between dienophiles and tetrazines.

IEDDA is another well-known click chemical reaction system without copper catalysis, which was initially reported by Fox and Blackman et al. in 2008 [58]. The Diels–Alder cycloaddition with high-tension cycloalkenes or cycloalkynes can be performed with tetrazine structure components (Fig. 5). The IEDDA reaction is known for its remarkable speed as a bioorthogonal reaction, making it suitable for in vivo and cellular applications [59]. Importantly, the rate of the reaction can be regulated by selecting different double bonds. For example, the rate constant of cyclopropene is 10^0^ to 10^4^ M^−1^ s^−1^, while the rate constant of 8-membered cycloolefin is approximately 10^6^ M^−1^ s^−1^ [60,61]. The IEDDA reaction was first employed for the encoding and fluorescent labeling of unnatural amino acids [62,63]. It is worth noting that when the fluorescent groups are attached to the tetrazine, their fluorescence is not strong due to the transfer of fluorescence resonance energy. However, after the cycloaddition reaction was completed, the structure of tetrazine was destroyed and the fluorescence was greatly enhanced [64,65]. Therefore, some tetrazine-decorated fluorescent molecules were applied in the field of life-cell imaging.

### Light-catalyzed bioorthogonal reactions

**Fig. 6.**
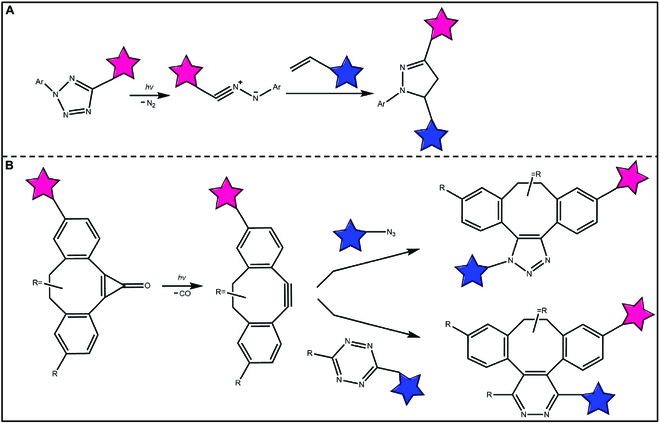
Light-catalyzed bioorthogonal reactions. (A) Photo-activated tetrazole-alkene 1,3-dipolar cycloaddition reaction. (B) Photo-triggered alkyne-azide cycloaddition reaction.

Some small molecules absorb ultraviolet (UV) or visible light, causing outer electron transition to the excited state [66–68]. During this process, they can overcome the activation energy of the reaction, enabling certain chemical reactions that are difficult to occur under normal conditions smoothly. On the other hand, due to the highly controllable light, photochemical reactions offer a possibility to precisely regulate bioorthogonal reactions temporally and spatially. A typical example of this is the addition reaction of 2,5-diphenyltetrazole and crotonic acid analogs under UV light [69]. In another part, tetrazole can be converted to 1,3-dipole by nitrogen removal under light, and then quickly coupled with olefin through cycloaddition (Fig. 6) [70]. Meanwhile, cycloallenone can strip a molecule of CO under light to obtain a high-tension 8-membered ring, thus participating in the subsequent cycloaddition reaction [71]. Therefore, the occurrence of bioorthogonal reactions can be controlled by light, to realize the “light control click chemistry” reactions.

### Metal-catalyzed bioorthogonal reactions

#### Cu(I) catalyzed azide–alkyne cycloadditions

**Fig. 7.**
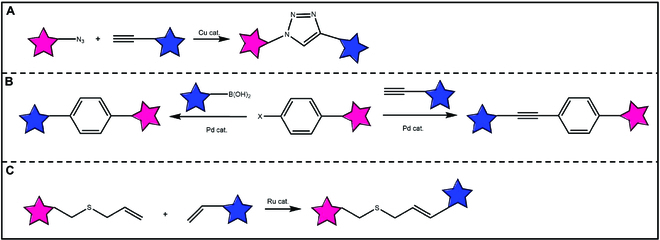
(A) The CuAAC. The click reaction between azide and alkyne generates triazole products by using a Cu-based catalytic route. (B) Suzuki–Miyaura cross-coupling (left) and copper-free Sonogashira cross-coupling (right) catalyzed by Pd. (C) Ru-mediated olefin cross-metathesis involving the use of allyl chalcogen-based privileged substrates.

Among the bioorthogonal reactions, CuAAC and SPAAC are widely recognized as the most prominent. In 2002, Tornøe et al. [72] and Rostovtsev et al. [73] independently reported that the rate constant of the Huisgen 1,3-dipole cycloaddition reaction based on azide–alkyne can be increased by a factor of 10^6^ under the catalysis of Cu(I) salts (Fig. 7A). Mechanism studies have shown that the formation of copper alkyne and dinuclear Cu(I) intermediates greatly improves the reaction efficiency [74]. It is worth noting that the formation of 1,4-bisubic triazole-linked units through the CuAAC reaction is similar to amide bonds in size and geometry, but it exhibits higher thermal and chemical stability compared to amide bonds [75]. Additionally, the CuAAC reaction can be carried out rapidly in the aqueous phase, usually does not generate any by-products, and is compatible with most functional groups [76]. Therefore, CuAAC reactions have a wide range of applications in materials science, bio-coupling, polymeric chemistry, pharmaceutical chemistry, and proteomics [77–79].

#### Other metal-catalyzed bioorthogonal reactions

Other traditional metals such as palladium (Pd), ruthenium (Ru), gold (Au), iron (Fe), iridium (Ir), etc. can also be utilized to catalyze bioorthogonal reactions (Fig. 7B and C) [80–84]. By Pd(0) catalyst, protective groups such as propargyl, allyl formate bond, and propargyl formate bond can be selectively cleaved for the activation of fluorescent and drug molecules [85]. Besides, Pd(0) can be used to generate bioactive drugs by C–C bond coupling reactions [86]. Pd(II) catalyst is mainly used for the cleavage of propargyl formate bond and bialene bond [87,88]. When utilizing Ru as a catalyst, the main mechanism involves the removal of the protective group of the allyl formate bond, thereby activating the fluorescent and drug molecules [89]. Au(III) can catalyze the propargyl ester bond into 5- or 6-membered rings, inducing the non-fluorescent substrate to become a fluorescent one [90]. Hence, this reaction could be applied to the fluorescence labeling of cells. Meanwhile, the Au catalyst can trigger the break of the propargyl ester bond, acetylene ether bond, propargyl, and other groups, so that the active groups of drugs are released [91], which can be used for the activation of drugs.

## Bioorthogonal Reactions for Biomedical Applications

Because of its numerous superiorities such as simplicity, effectiveness, specificity, etc., the bioorthogonal reaction as a very important chemical biology tool, has attracted extensive attention and has also been widely used in many fields such as biomedicine, biological materials, and cancer diagnosis and treatment [76,92,93]. For example, by using genetic engineering and corresponding means, one of the functional groups can be integrated into a specific biomacromolecule in advance, which can subsequently interact with the other groups connected to a fluorescent group [94]. Thus, imaging at the level of living cells can be realized to study the location and function of the target biomacromolecule. Similarly, a functional group capable of bioorthogonal reaction can be integrated at a specific location of antibody macromolecules using genetic engineering, and then a toxin or nuclide molecule can be attached to the antibody to obtain a site-modified antibody-conjugated drug [95]. If the 2 functional groups involved in a bioorthogonal reaction are positioned at the ends of the 2 polymer segments, they can be easily connected, resulting in a polymer with a higher molecular weight [96]. In this section, the biomedical applications of bioorthogonal reactions will be introduced in detail.

### Bio-labeling

The exploration and understanding of the nature of life pose fundamental scientific challenges that have fascinated human beings for centuries. Among these challenges, the study of life activities through the labeling of biological targets emerges as an important research approach [97]. With the rapid development of biotechnology, the observation of biological systems is no longer limited to static observation, but gradually turned to real-time dynamic monitoring. In this context, an accurate and efficient mark of biological targets is the key parameter. Bioorthogonal chemistry and click chemistry are outstanding in this field because of their advantages of high reaction rates and efficiency, and high stereoselectivity. Together with isotope labeling (1943 Nobel Prize in Chemistry) [98], fluorescent protein labeling (2008 Nobel Prize in Chemistry) [99,100], single-molecule super-resolution imaging (2014 Nobel Prize in Chemistry) [101,102], and other technologies, it has been utilized in the labeling of proteins, lipids, sugars, organelles, and other biological targets in this field.

#### In vitro labeling

**Fig. 8.**
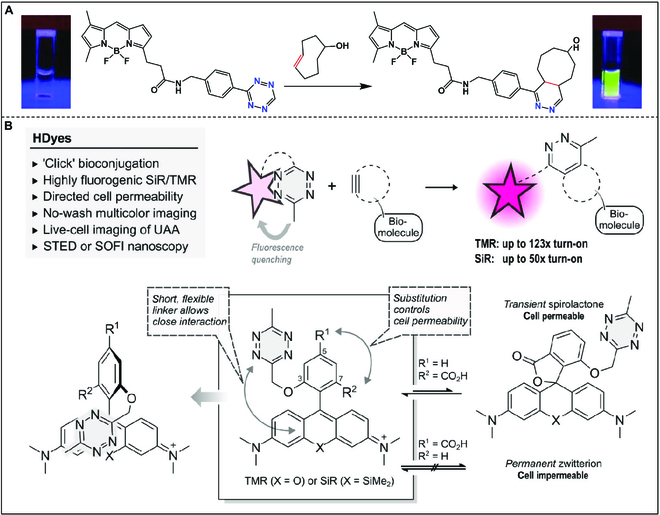
(A) Tetrazine–BODIPY reacts rapidly with *trans*-cyclooctene in an IEDDA cycloaddition to form isomeric dihydropyrazine products, resulting in a sharp increase in fluorescent activity. Reproduced with permission [103]. Copyright 2010, Wiley-VCH GmbH. (B) Design rationale for proximity-based quenched fluorogenic probes. Tetrazine dyes can be linked to dienophile-modified biomolecules, leading to enhanced fluorescence intensity. Reproduced with permission [104]. Copyright 2021, American Chemical Society.

Bioorthogonal reactions can be employed to carry out in vitro cell studies on the labeled targets, promoting mechanism research and the development of novel drugs. For example, Devaraj et al. [103] successfully implemented fluorescent labeling of tubulin in PtK2 cells using the IEDDA reaction (Fig. 8A). Tubulin is a vital component of the eukaryotic cytoskeleton, which is involved in many cellular functions such as structural support, intracellular transport, and DNA separation. Currently, several anti-cancer drugs targeting tubulin have been approved, such as the representative Paclitaxel and Vinblastine.

In another case, Werther et al. [104] report on a new class of tetrazine-silyl rhodamine couplings, HDyes (“Heidelberg Dyes”), for biological orthogonal labeling of both intracellular and extracellular targets (Fig. 8B). The fluorescence of the HDyes molecule is efficiently quenched, but the fluorescence is enhanced after the IEDDA reaction occurs with the alkynyl-containing biomolecules. In general, effective fluorescence quenching of red-shifted fluorophores in tetrazine fluorescent dyes is difficult to achieve. However, the author introduced the tetrazine structure into the ortho-site of the benzene ring, and its short and flexible methoxy-group connection mode would lead to the spatial accumulation of the fluorescence structure, and the sufficiently close distance between the tetrazine and the fluorophore would facilitate the fluorescence quenching of the molecule. In addition, the cell permeability of the compound was controlled by changing the position of substituents on the benzene ring. The results show that the o-carboxyl-substituted HDyes can form transient spironolactone without charge, which has high cell permeability. In contrast, para-carboxyl-substituted HDyes is a permanent amphoteric dye that is not capable of spirolactone and thus has no cellular permeability. They investigated the photophysical properties of HDyes and evaluated their applicability in bioconjugation, and successfully used them to label enhanced green fluorescent protein (EGFP) in vitro. In cell permeability verification experiments, HDyes dye can perform live cell bicolor imaging on intracellular and extracellular targets under leave-in conditions, and has good specific labeling. In addition, the compound has been successfully applied to stimulated emission depletion microscopy and super-resolution optical fluctuation imaging. Due to its excellent photophysical properties, improved signal-to-noise ratio, and small labeling size, the molecule is expected to be widely used in the field of bioorthogonal labeling super-resolution microscopy.

#### In vivo labeling

**Fig. 9.**
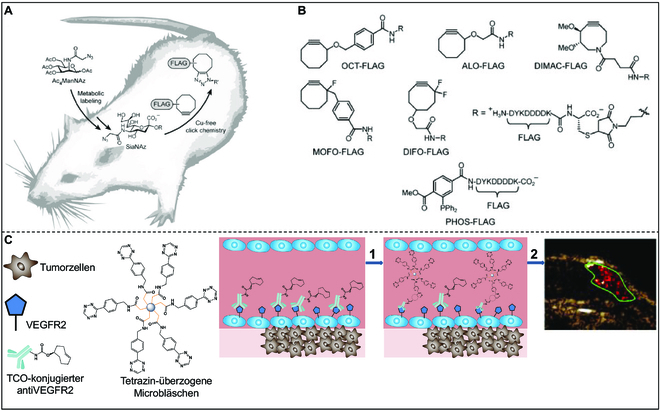
(A) Mice were injected with Ac_4_ManNAz once a day for 1 week in order to metabolize the glycans with SiaNAz. Then, the mice were injected with a cyclocyanine–FLAG conjugate, which was used to covalently label azide polysaccharides in vivo. (B) The FLAG conjugate panels were used in this study. Reproduced with permission [107]. Copyright 2010, National Academy of Sciences. (C) MBs were localized to tumor cells through pretargeting and bioorthogonal chemistry between tetrazine-modified microvesicles and the TCO-functionalized intravascular target (VEGFR2). Reproduced with permission [108]. Copyright 2014, Wiley-VCH GmbH.

The complex environment in vivo has put forward higher requirements for accurate labeling targets [105,106]. Bioorthogonal reactions have been widely used in in vivo labeling due to their unique advantages in reaction selectivity, reaction conversion efficiency, and reaction rate. Chang et al. [107] studied o-acetylglucosamine (O-GlcNAc) in a mouse model by utilizing the SPACC reaction (Fig. 9A and B). O-GlcNAcylation is a common post-translational protein modifier that is closely related to central nervous system degenerative diseases and type II diabetes. Bertozzi started his research by injecting peracetylated *N*-azidoacetylmannosamine (Ac_4_ManNAz) into a mouse. Acetyl-modified Ac_4_ManNAz increased the cell membrane transmobility of non-natural sugar molecules. After entering cells, intracellular carboxylesterase could remove the acetyl group of Ac_4_ManNAz to produce azido-labeled SiaNAz (a kind of sugar on the cell membrane surface). Next, the mouse was injected with FLAG (Asp-Tyr-Lys-Asp-Asp-Asp-Asp-Lys) polypeptide modified with alkynes, so that SiaNAz could be labeled by SPACC bioorthogonal chemical reaction. Thus, by using fluorescent molecule-labeled FLAG monoclonal antibody, the distribution and metabolism of SicNAz in different tissues and organs of mice can be monitored through fluorescence signals.

In another case, the team of John F. Valliant, from McMaster University in Canada, used bioorthogonal chemistry to modify ultrasound contrast agents, realizing accurate ultrasound observation of tumor tissue (Fig. 9C) [108]. In this study, *trans*-cyclooctene (TCO) was chemically modified on the vascular endothelial growth factor receptor 2 (VEGFR2) antibody, and multiple tetrazine groups were modified on the surface of hollow microbubbles (MBs, an ultrasound contrast agent). The VEGFR2 can target tumor tissue, while the MBs connect the VEGFR2 via IEDDA bioorthogonal chemical reactions, making accurate ultrasound observation of tumor tissue possible.

**Fig. 10.**
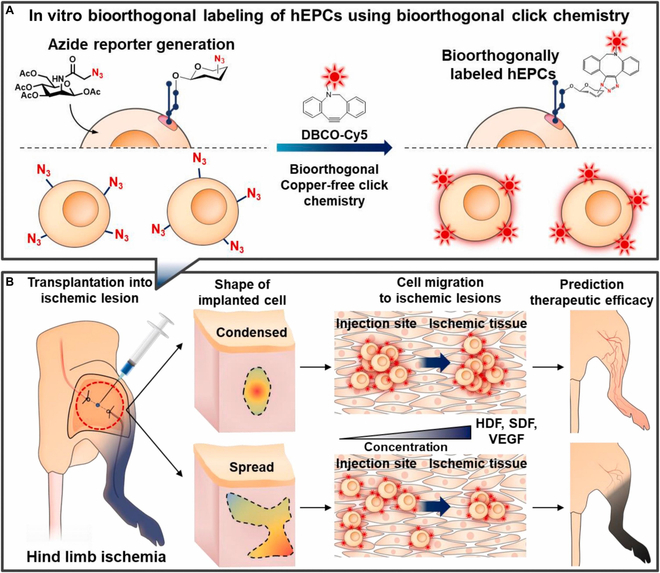
Schematic representation for assessing the therapeutic effectiveness of bioorthogonally labeled hEPCs in a hind limb ischemia model through non-invasive FMT imaging. (A) In the process of bioorthogonal labeling, unnatural azide groups were introduced on the hEPC surface through Ac_4_ManNAz pretreatment. Subsequently, these azide groups on hEPCs are labeled with DBCO-Cy5 using bioorthogonal copper-free click chemistry in vitro. (B) Subsequently, in an in vivo hind limb ischemia model, non-invasive FMT was used to image the bioorthogonally labeled hEPCs (Cy5-hEPCs) over a period of 28 days. Reproduced with permission [109]. Copyright 2021, Elsevier.

Kim et al. devised a copper-free click chemistry-based system for labeling and tracking endothelial progenitor cells (EPCs) to predict in vivo therapeutic efficacy in a hind limb ischemia model (Fig. 10) [109]. Human embryonic stem cell-derived EPCs are employed in the treatment of ischemic diseases owing to their unipotent ability to differentiate into mature endothelial cells, promoting angiogenesis and vasculogenesis. Significantly, EPCs exert a paracrine effect that aids in neovascularization by secreting growth factors such as vascular endothelial growth factor, stromal cell-derived factor-1α, and angiopoietin-1. Before transplanting hMSCs into the ischemic lesion, they were pretreated with Ac4ManNAz to introduce azide groups onto their surfaces. Subsequently, dibenzocycloecten (DBCO)-Cy5 was conjugated to these azide groups using SPAAC. Cy5-hEPCs labeled with DBCO-Cy5 displayed a robust Cy5 signal and showed no notable toxicity or functional abnormalities in terms of oxygen consumption, differentiation, or paracrine effects. Moreover, the migration of the near-infrared fluorescence signal from Cy5-hEPCs could be tracked using a fluorescence molecular tomography (FMT) imaging system for 28 days following transplantation into the hind limb ischemic lesion. Intriguingly, the therapeutic outcomes, encompassing blood reperfusion and neovascularization in the ischemic lesion exhibited evident variations based on the initial transplantation form of Cy5-hEPCs. Limb loss occurred in only 20% of the mice when Cy5-hEPCs were initially transplanted in a condensed form. In contrast, 30% and 40% of the mice lost their limbs when treated with hEPCs or Cy5-hEPCs that initially exhibited a spread shape, respectively. These findings suggest a close correlation between the therapeutic effects of hEPCs and their initial transplantation shape, highlighting the potential utility of monitoring the initial transplantation shape for predicting therapeutic outcomes.

#### Ex vivo labeling

**Fig. 11.**
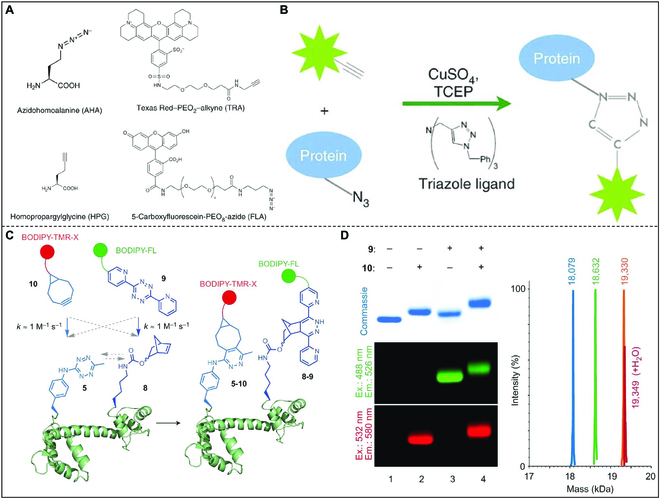
(A) The chemical structures of the modified amino acids AHA and HPG, and 2 fluorescent labels TRA and FLA used to visualize the newly synthesized protein. (B) Scheme illustration of the CuAAC principle. Reproduced with permission [110]. Copyright 2010, Springer Nature. (C) Strategy for protein double-labeling via IEDDA reactions. (D) Quantitative and site-specific double-labeling of proteins. Reproduced with permission [111]. Copyright 2014, Springer Nature.

Ex vivo detection can provide valuable information about tissue development, disease diagnosis, disease surveillance, and other aspects. Bioorthogonal chemistry is also useful in this respect. A team led by Erin M. Schuman studied isolated rat primary hippocampal neurons with CuAAC click chemistry for observation of new protein synthesis in situ using fluorescence microscopy (Fig. 11A and B) [110]. Based on the core chemical properties of bioorthogonal noncanonical amino acid tagging, 2 different fluorescent labels (Texas Red-PEO_2_-alkyne [TRA]; 5′-carboxyluciferin-PEO_8_-azide [FLA]) can be conjugated to azidohomoalanine (AHA) or homopropargylglycine (HPG)-carrying proteins by bioorthogonal chemistry. They named this visualization method FUNCAT (fluorescent noncanonical amino acid tagging), which allows the in situ detection of newly synthesized broad-spectrum neuronal proteins.

Prof. Jason W. Chin and co-workers cleverly designed 2 pairs of Forster resonance energy transfer (FRET) donor molecules and acceptor molecules based on the bioorthogonal chemical characteristics of IEDDA to study the conformation of calmodulin (Fig. 11C and D) [111]. BODIPY-FL (donor) and BODIPY-TMR-X (acceptor) were selected as FRET probes due to the substantial overlap between their emission and excitation spectra. Through meticulous characterization of the kinetics of the IEDDA reaction, a strategy was devised to encode tetrazine and norbornene. These compounds do not react with each other in proteins, but they can be efficiently labeled using complementary diclonal acetylene and electron-deficient tetrazine. This work presents a pioneering modular approach for installing various probe pairs into any genetic control site within a protein while maintaining physiological temperature, pressure, and pH conditions. The developed modular approach will be highly valuable for labeling proteins with a diverse range of fluorophores, facilitating integral and single-molecule FRET experiments, as well as other biophysical measurements.

Bioorthogonal reactions enable precise biomolecule labeling and tracking within living systems. These reactions are recognized for their exceptional selectivity, minimal disruption to native biological processes, and compatibility with living organisms. Despite their immense utility in bio-labeling and biomolecule studies within living systems, certain limitations still need to be noticed. While bioorthogonal reactions are designed to be biocompatible, not all reactants and conditions are equally well-tolerated by living cells or organisms. Toxicity, cellular stress, or unwanted side effects can occur, particularly when using certain catalysts or reactants. Besides, bioorthogonal bioconjugates may not always be as stable as desired in biological environments. Factors such as proteolytic degradation and clearance mechanisms could impact the longevity of the labeled biomolecules, potentially leading to loss of signal over time. Some bioorthogonal reagents and probes can be expensive or may have limited commercial availability, which can be a barrier to their use in certain research settings. Researchers should carefully consider these limitations when designing experiments and choose bioorthogonal reactions and conditions that are best suited to their specific research goals and biological systems.

### Nucleic acid functionalization

**Fig. 12.**
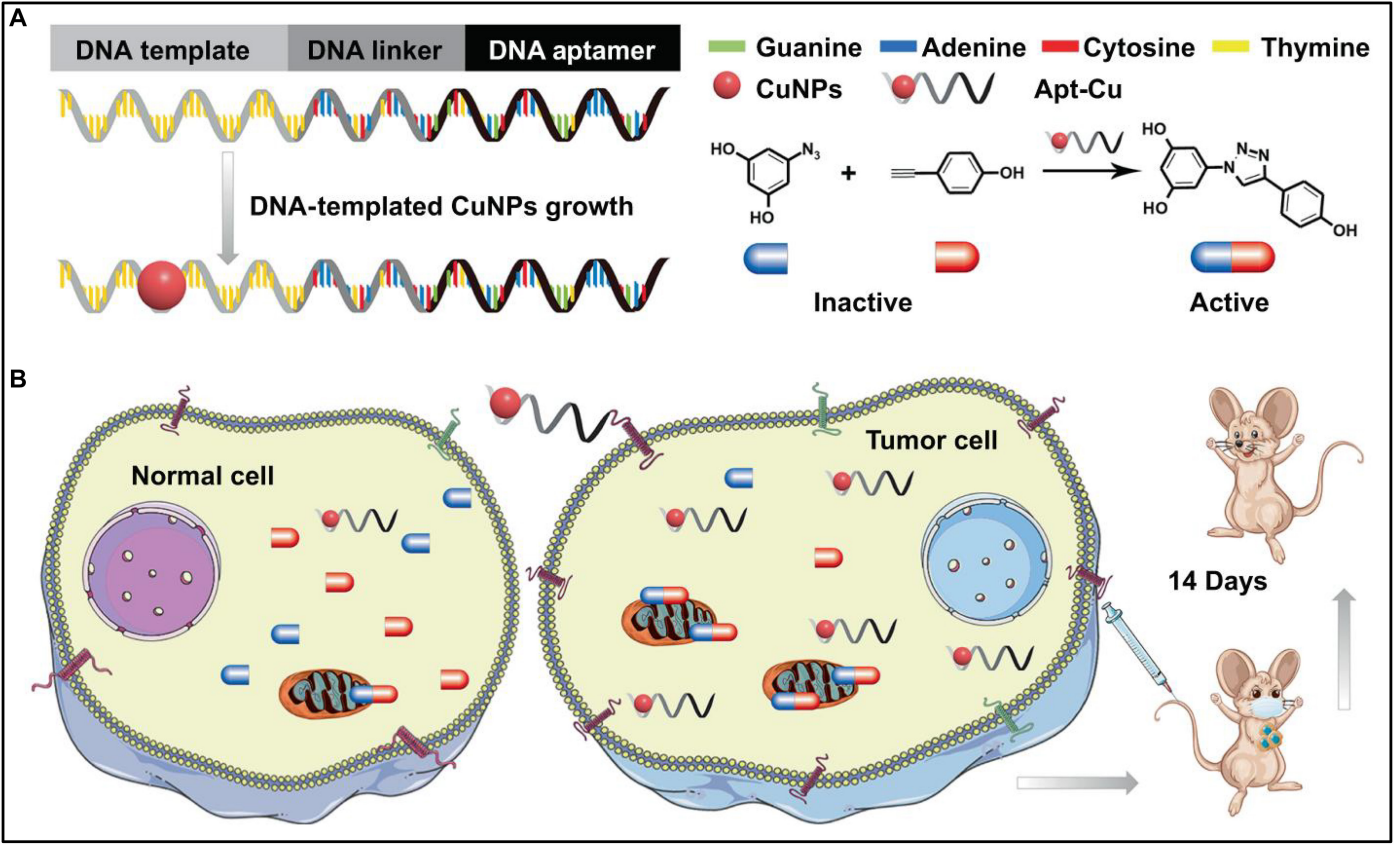
(A) Design and synthesis of DNA-templated CuNPs as bioorthogonal catalysts. (B) Aptamer-mediated CuNPs were used for cell-specific identification and targeted cancer therapy in vivo. Reproduced with permission [112]. Copyright 2022, Springer Nature.

The nucleic acid functionalization based on bioorthogonal chemistry is helpful to the flexible design and to extend the versatility of nucleic acid. For example, You et al. [112] designed a DNA bioorthogonal nanocatalyst for tumor therapy. As shown in Fig. 12, the single-stranded DNA nanocatalyst comprises 3 domains. One of the domains is a thymine-rich sequence, which serves as a template for the formation of copper nanoparticles (CuNPs). Another domain consists of aptamers specifically targeting cancer cells, such as mucin 1 (MUC1), which exhibits unique and abundant expression on the surface of adenocarcinoma cells. The third domain is a 15-base chain that links various lengths of thymine-rich regions to different types of aptamers. The DNA strand serves as both the structural foundation of the nanocarrier and the template for the formation of CuNPs. Additionally, it exhibits specific recognition toward target cells. The resulting nanocatalysts demonstrate favorable biocompatibility and exceptional catalytic conversion efficiency, and exhibit type-specific cell recognition in vitro. The simple change of different aptamers can be realized through the programmability of nucleic acid, to achieve flexible tumor targeting, indicating that this is a promising approach for personalized cancer therapy.

**Fig. 13.**
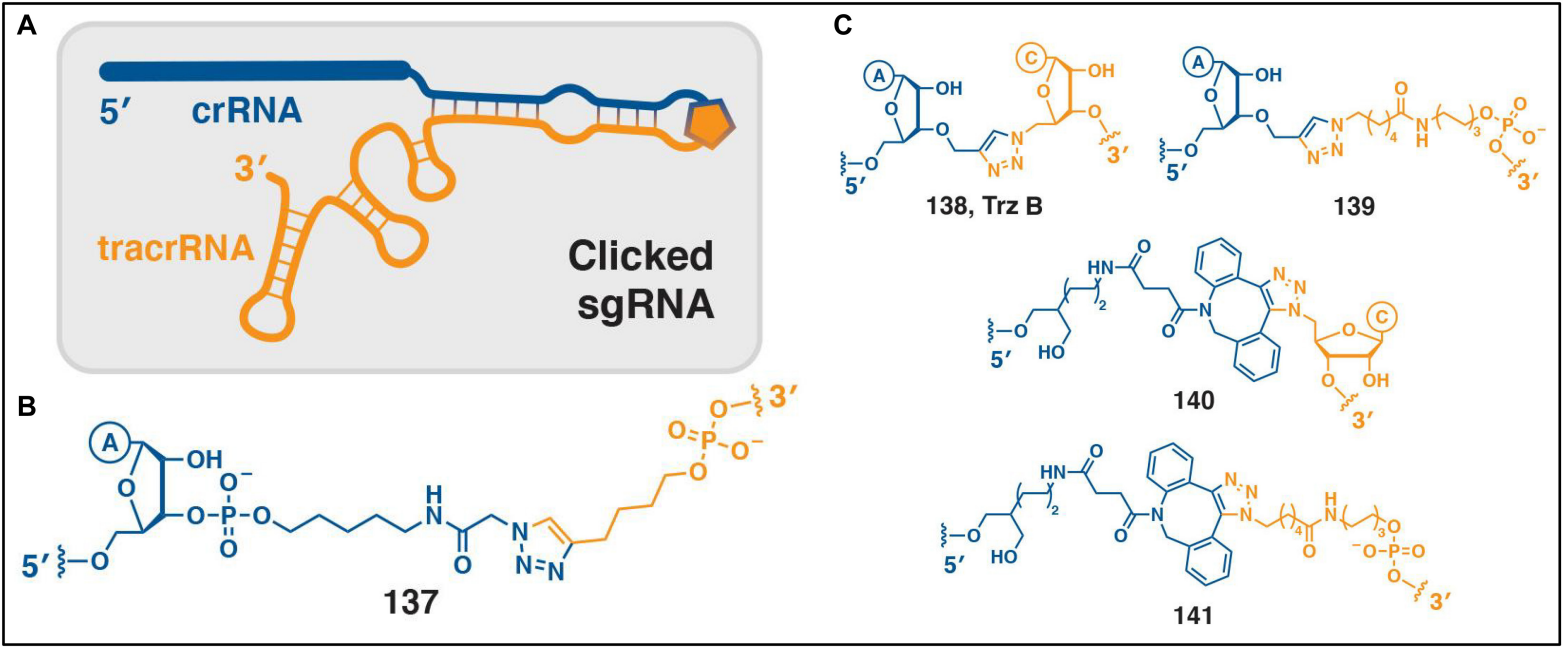
(A) A 3′-alkyne (or azide) modified crRNA and a 5′-azide (or alkyne) functionalized Cas9-binding RNA are clicked together in the upper stem to produce a chemically linked sgRNA structure in which a natural phosphate bond is replaced by triazole. (B) Type of triazole linkage used by He and colleagues. Reproduced with permission [114]. Copyright 2016, Wiley-VCH GmbH. (C) Type of triazole linkages used by Brown and colleagues. Reproduced with permission [115]. Copyright 2019, Springer Nature.

In addition, the modularization provided by the azide–alkyne cycloaddition can be used as a nucleic acid assembly method for biological and nanotechnology applications. Strategies have been developed for the chemosynthesis of genomic DNA fragments, especially those incorporating modified and epigenetic bases, and have been formulated in vitro and in cells. Genes assembled using this approach exhibit functionality in both prokaryotic and eukaryotic systems (Fig. 13) [113]. Furthermore, the click linkage of oligonucleotides is a valuable technique for generating antisense oligonucleotides. In this scenario, the inclusion of a triazole moiety enhanced the stability of the oligonucleotide against nuclease degradation and decreased its anionic charge, potentially facilitating cellular uptake. Additionally, chemosynthesis and click-linking techniques have been employed to incorporate base modifications and nucleoside derivatives, such as locked-in nucleic acids and G-clamps, aimed at enhancing target binding and improving mismatch sensitivity. Moreover, the utilization of bioorthogonal chemistry in the highly targeted and biologically noteworthy domain of CRISPR-Cas gene editing enables the rapid and high-throughput production of sgRNA libraries. He et al. [114] successfully prepared bimolecular-directed RNA systems by employing CuAAC chemistry to combine 5'-hexyne tracrRNA (65-mer) with a 3'-azide crRNA component (34-mer). Taemaitree et al. [115] also modified the same ring with triazole bonds of different lengths, conjugated 37-mer crRNA and 66-mer tracrRNA to form sgRNA constructs via SPAAC or CuAAC reactions.

### Drug discovery

**Fig. 14.**
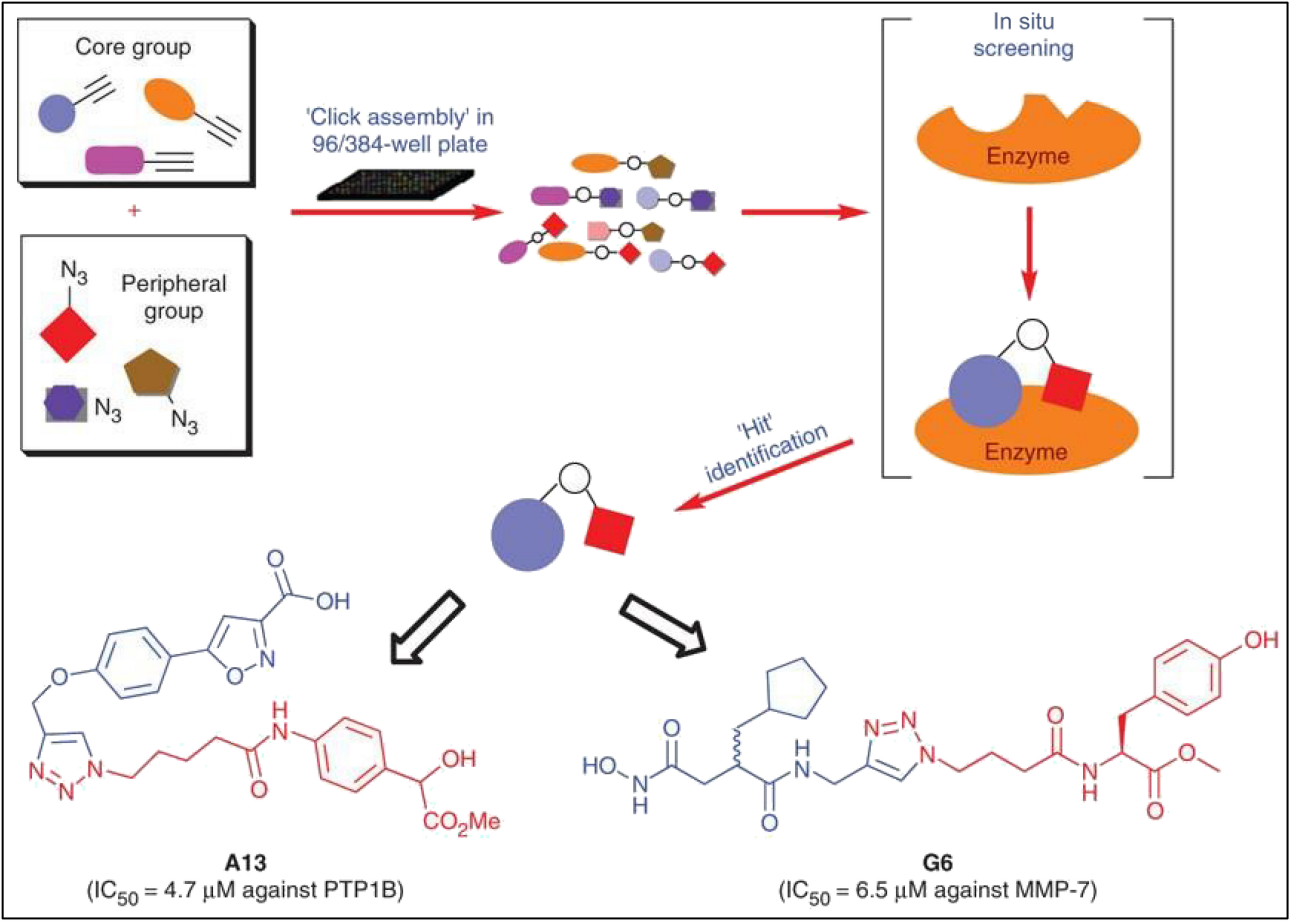
Inhibitors were discovered by click chemistry and then directly screened for 2 major enzymes (PTPs and MMPs). Reproduced with permission [117]. Copyright 2007, Springer Nature.

Bioorthogonal chemistry specializes in the efficient linking of molecular fragments and is a practical approach in the field of high-throughput screening (HTS) and fragment-based drug discovery (FBDD) [116]. In a typical example, Srinivasan et al. [117] have tried to apply bioorthogonal chemistry to the HTS of drugs (Fig. 14). In their study, 2 enzymes, protein tyrosine phosphatase (PTP) and matrix metalloprotease (MMP), which are closely related to physiological activities, were selected as targets. The 2 molecular fragments are efficiently linked by the bioorthogonal reaction to screen for corresponding enzyme inhibitors. PTP is closely related to cell proliferation and differentiation. MMP is closely associated with embryogenesis, normal tissue remodeling, wound healing, and angiogenesis. Because of its remarkable modularity and exceptional reactivity, bioorthogonal chemistry represents one of the most viable approaches for the development of fragment-based inhibitors. By harnessing the capabilities of bioorthogonal chemistry, it becomes possible to design closely related yet fragmented prodrug compositions that exhibit specific interactions with molecular targets, including proteins or genes. This innovative approach holds promise for personalized therapy.

**Fig. 15.**
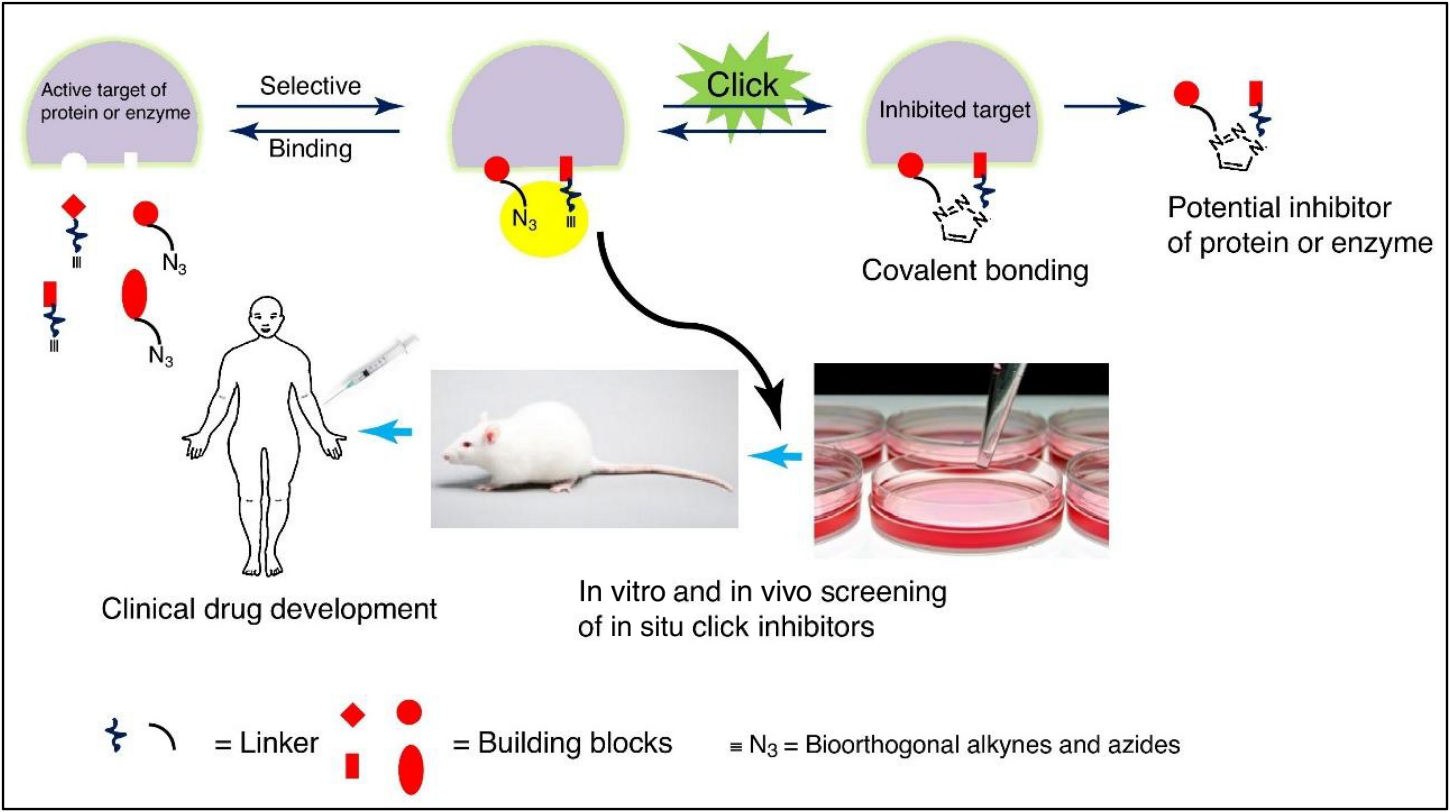
Schematic diagram of in situ click chemistry used to develop enzyme or protein inhibitors and drug development. Reproduced with permission [118]. Copyright 2017, Elsevier.

The integration of bioorthogonal chemistry with high-throughput enzyme assay techniques, including microarrays, has revolutionized lead discovery and lead optimization in the field of drug development. Owing to the efficiency and water compatibility of bioorthogonal reactions, the assembled products can undergo direct screening for inhibition without the need for purification. Small molecule libraries synthesized through bioorthogonal chemistry have demonstrated the successful generation of distinctive inhibitors and active guide fingerprints for significant enzymes, thus facilitating the identification and characterization of novel enzyme subclasses (Fig. 15) [118].

**Fig. 16.**
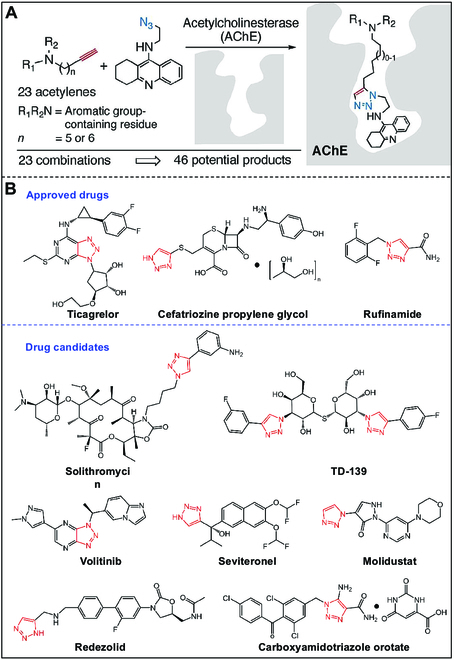
(A) In situ click chemistry screening for AChE inhibitors. Reproduced with permission [119]. Copyright 2005, American Chemical Society. (B) Approved drugs or drug candidates containing 1,2,3-triazole structure. Reproduced with permission [120]. Copyright 2019, Taylor & Francis Ltd.

Professor Sharpless, who came up with the idea of click chemistry and CuAAC reaction, is also experimenting with click chemistry in the field of FBDD. They selected acetylcholinesterase (AchE) as a target and found a highly active AchE inhibitor by connecting 2 molecules in situ in a pocket (Fig. 16A) [119]. The investigation of structure–activity relationships of drug molecules is a fundamental area of study in pharmaceutical chemistry. Notably, the triazole structure, which can be synthesized using the classical CuAAC chemical reaction, serves as a ubiquitous core motif in numerous pharmaceutical compounds. Recent studies have demonstrated that the triazole structure can have diverse effects on drug molecules, serving as a linking unit between the pharmacophore and other groups, targeting conjugates, or drug carriers, and as a molecular conformation adjustment unit, among other functions, including its role as a specific biological functional group. Several approved or prospective drug molecules contain the structure of triazole (Fig. 16B) [120].

Bioorthogonal reactions are increasingly used in the field of drug discovery, including target confirmation (such as phenotypic screening), study of drug–target binding, and selective screening of lead compounds [120]. It is well known that one of the toughest challenges in medicinal chemistry is to rationalize and accelerate the discovery and optimization of lead compounds [121]. To solve this problem, a multifunctional chemistry toolbox is essential. The bioorthogonal reaction has a powerful energy drive to ensure that the starting compound reacts quickly and efficiently, and no harmful substances are produced under physiological conditions, making this transformation a perfect model of bioorthogonality. Click chemistry and bioorthogonal reactions are important components of the medicinal chemistry toolbox and offer substantial advantages to medicinal chemists in overcoming the limitations of useful chemical synthesis, increasing yields, and improving the quality of their compound libraries.

### Drug activation

In addition to drug discovery, bioorthogonal reactions are often applied in drug activation to achieve better disease treatment [122]. The activation process of drugs is recognized to be influenced by diverse stimuli, encompassing both exogenous factors (e.g., temperature [123], light [124–126], ultrasound [127–129], magnetic field [130], and electric field [131]) and endogenous factors (such as hypoxic environments [132,133], pH [134], glutathione [135], reactive oxygen species [136,137], and enzymes [138]) [139,140]. The bioorthogonal reaction of the broken bond type exhibits high reaction efficiency and specificity and can achieve the controlled release of drugs, thereby enhancing treatment efficacy and reducing the toxic side effects.

**Fig. 17.**
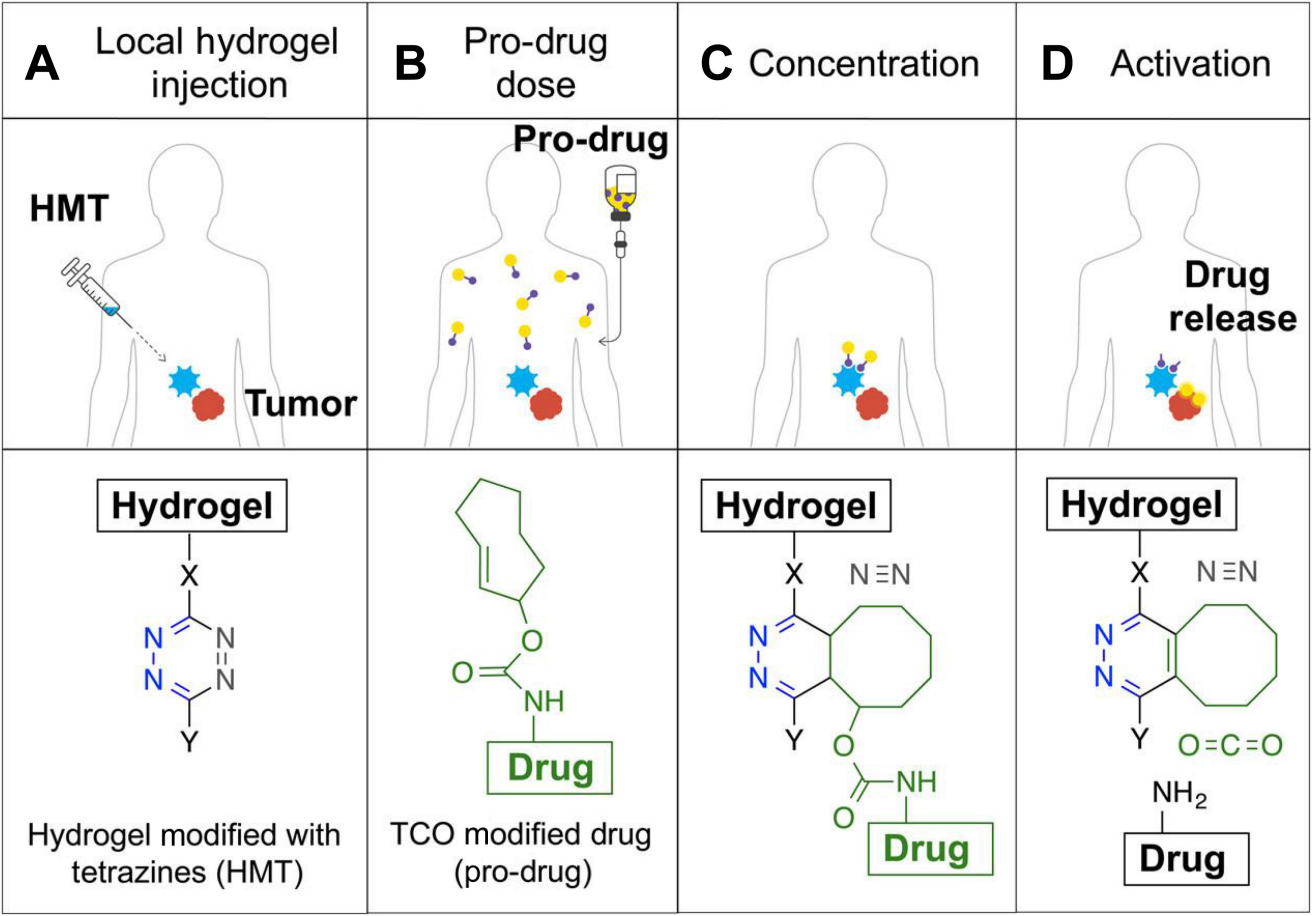
In vivo bioorthogonal chemistry for the concentration and activation of systemic prodrugs. (A) A hydrogel, modified with tetrazine (Tz), is injected into the specific treatment area. (B) The patient is administered a pro-drug covalently modified with a TCO carbamate. (C) When the pro-drug comes into contact with the hydrogel, a rapid cycloaddition reaction occurs, increasing the drug concentration at the target site and releasing nitrogen molecules. (D) Subsequently, the resulting cycloadduct undergoes in vivo isomerization, leading to the decomposition of the self-immolable carbamate linker. This process releases carbon dioxide and, most importantly, the drug at the local site, enabling it to perform its therapeutic function. Reproduced with permission [141]. Copyright 2016, American Chemical Society.

Recently, Shasqi Bio Inc. has been actively engaged in the development of the Click Activated Protodrugs Against Cancer (CAPAC) platform, which utilizes bioorthogonal reactions, particularly the IEDDA reaction, for applications in patients. (Fig. 17) [141]. They are utilizing SQ3370 to demonstrate the clinical effectiveness of this platform, marking the first instance of a bioorthogonal chemistry reaction being performed in a patient [142]. Such an approach aims to activate cancer drugs at the tumor site and reduce systemic toxicity. The procedure involves the local injection of an alginate saline gel that has been modified with a click chemical handle, specifically tetrazine, at the tumor site. Subsequently, an intravenous prodrug is administered, which possesses a complementary handle, for example, TCO-modified doxorubicin (DOX). The prodrug remains inert within the body until it encounters the hydrogel during metabolic processes. At this point, a click chemical reaction is triggered, leading to the release of the active drug at the tumor site. This localized drug activation approach effectively reduces off-target exposure and enables the administration of a highly concentrated dose of the drug at the tumor site. In a xenograft model, where treatment was monitored for over 100 days, the median tumor size remained unchanged in the DOX prodrug treatment group. In contrast, the control group experienced a rapid rebound in tumor size on day 50. This compelling outcome demonstrates the potential of this approach in selectively targeting and treating tumors while minimizing systemic effects. Meanwhile, the body weight of the DOX prodrug group remained stable, while it declined in the control group throughout treatment.

**Fig. 18.**
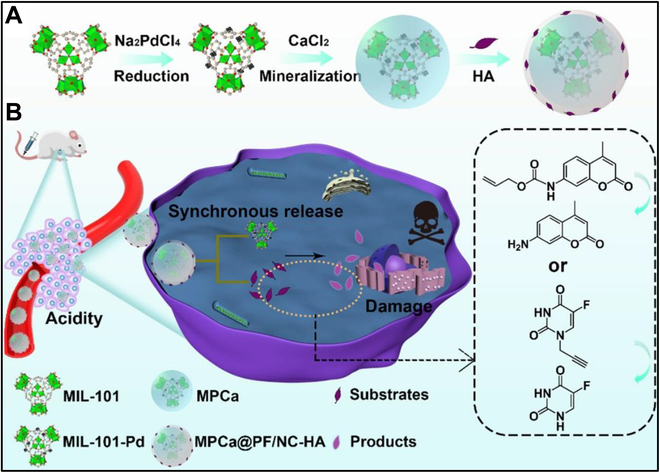
(A) Illustration of the synthetic process of nanoparticles. (B) Bioorthogonal reactions catalyzed by the All-in-One system. Reproduced with permission [143]. Copyright 2022, American Chemical Society.

Moreover, Wu et al. [143] reported an “All-in-One” system consisting of bioorthogonal catalyst Pd nanoparticles and prodrug molecules 5-fluoro-1-propargyluracil (Pro-5FU) (Fig. 18). The Pd nanoparticles were supported in a metal-organic framework (MIL-101-Pd) and mineralized with a CaCO_3_ layer to separate them from prodrug molecules. Under acidic conditions of tumor cells, the decomposition of CaCO_3_ promotes the activation of Pro-5FU catalyzed by Pd nanoparticles through bioorthogonal reaction, thus effectively killing tumor cells. This research offers a universal nanosystem for bioorthogonal drug activation that could implement prodrug release and on-demand in situ drug production in tumors.

### Synthesis of antibody–drug conjugates

**Fig. 19.**
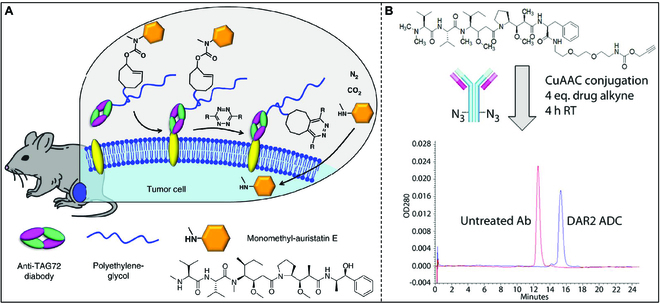
(A) Triggered drug release using “click-to-release” chemistry in vivo: on-tumor liberation of a cell-permeable drug from a *trans*-cyclooctene-linked ADC following systemic administration of a tetrazine activator. Reproduced with permission [147]. Copyright 2018, Springer Nature. (B) Azide–alkyne linkage for the generation of ADCs. Reproduced with permission [149]. Copyright 2015, American Chemical Society.

In 1913, the Nobel Prize-winning German scientist Paul Ehrlich first came up with the concept of a “Magic bullet”: a toxin on a carrier that could target cancer cells so precisely that it could kill them without harming normal cells [144,145]. Subsequently, antibody–drug conjugates (ADCs) emerged to realize the idea. Bioorthogonal chemistry, renowned for its proficient molecular binding capabilities, is frequently employed in the research and development of ADCs [146]. Tagworks Pharmaceuticals is currently engaged in the development of a bioorthogonal chemistry strategy aimed at addressing the limitations of ADCs in effectively targeting solid tumors. Traditional ADCs selectively target internalized receptors on cancer cells, inducing drug release by cleaving linkers within the intracellular environment. However, specific internalization receptor expression is observed in only a subset of patients with solid tumors. Herein, Tagworks Pharmaceuticals has developed a Click-to-Release approach to enable the application of ADC therapies to non-internalizing targets (Fig. 19A) [147]. The ADC targets non-internalized receptors that possess a joint susceptible only to bioorthogonal chemical reactions. Upon local enrichment of the ADC around tumor cells, an activator is administered systemically, engaging with the ADC joint outside tumor cells and facilitating drug release within the tumor microenvironment. Subsequently, the released drug is taken up by neighboring cancer cells and stromal cells that contribute to tumor support. The Click-to-Release approach broadens the application of ADCs by facilitating universal and precise temporal regulation of drug release within the tumor microenvironment. This advancement holds the potential to enhance therapeutic effectiveness in heterogeneous or poorly penetrating tumors by enabling more uniform drug delivery.

Researchers from Synaffix company have designed an efficient and cost-effective ADC conjugate method based on CuAAC, which can quickly synthesize ADCs. Based on this, they developed the GlycoConnect coupling technology, which can use natural glycosylation sites to achieve site-specific coupling [148]. The technique is based on 2 processes: first, enzyme remodeling (modification and labeling with azide), and then the linking of payloads based on copper-free click chemistry. The monoclonal antibody can be converted into a stable conjugated ADC in just a few days. MedImmune, a division of AstraZeneca that develops ADC drugs, has developed a family of ADC drug candidates through the CuAAC click chemical link. In the series of ADC candidate drugs, monoantimicrobials targeting HER2/neu (amember of the EGFR family) are modified with an azide group, and auristatin F (a cytotoxic tubulin inhibitor) is modified with a terminal alkyne (Fig. 19B) [149]. This technology has been expanded to generate fully functional and highly stable ADCs that exhibit optimized pharmacokinetic, biological, and biophysical properties.

The development of ADCs has advanced substantially in the past decade, driven by improvements in payloads, linkers, and coupling techniques [150,151]. Notably, linker design plays a pivotal role in governing ADC stability in systemic circulation and payload delivery efficiency in tumors, thereby influencing pharmacokinetics (PK), efficacy, and toxicity profiles [151,152]. Key linker parameters, including coupling chemistry, length, and steric hindrance, profoundly impact ADC performance [153]. The ideal linker should maintain stability in circulation while releasing cytotoxic payloads within tumors [154]. However, existing linkers often release payloads unpredictably, leading to off-target toxicity. Thus, in ADC design, precise adjustment of these linker parameters is imperative to strike a balance between stability and payload release efficiency, ensuring the desired therapeutic effect. Recent years have witnessed substantial progress in optimizing ADC structures and diversifying their mechanisms [155]. Novel cleavable linkers, especially bioorthogonal ones, hold promise for overcoming intracellular drug release limitations seen in traditional ADCs [156]. However, bioorthogonal cleavable linkers are predominantly explored in vitro. Challenges such as reaction efficiency, rate, substrate stability, biocompatibility, and ease of operation hinder their clinical application. Nevertheless, preliminary data from these innovative linkers show promise and are expected to significantly contribute to the rapid evolution of ADC drugs in the future.

### Synthesis of PROTACs

**Fig. 20.**
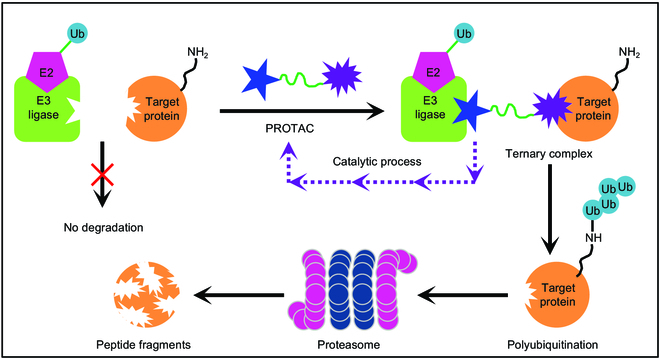
Schematic diagram of PROTAC mechanism.

PROTACs have attracted wide attention from researchers due to their ability to effectively degrade proteins for the treatment of many diseases [157,158]. PROTACs can bind to the protein of interest (POI) and the E3 ligase, forming a ternary complex. The POI can be brought close to the E3 ligase and labeled with ubiquitin. Subsequently, the target protein undergoes degradation through the ubiquitin–proteasome pathway (Fig. 20) [159,160]. In theory, a catalytic dose of PROTACs has the potential to induce the degradation of over 80% of cellular proteins, thereby transforming the target from being “non-druggable” to “druggable” [161]. Consequently, PROTAC technology holds great potential for overcoming drug resistance and targeting previously “non-druggable” entities. The success or failure of PROTAC research and development is contingent upon the linker selection. The length of the linker significantly impacts the degradation activity of PROTAC, with commonly employed linker lengths typically ranging from 4 to 15 carbon atoms (or heteroatoms) [162]. The effects of linker length on degradation activity vary depending on the target. Owing to its mild reaction conditions and high efficiency, bioorthogonal chemistry is frequently employed to link both ends of PROTAC molecules via the linker. Therefore, researchers have sought to integrate bioorthogonal chemistry with PROTAC, aiming to expedite the translation of PROTAC from a conceptual framework to practical application.

**Fig. 21.**
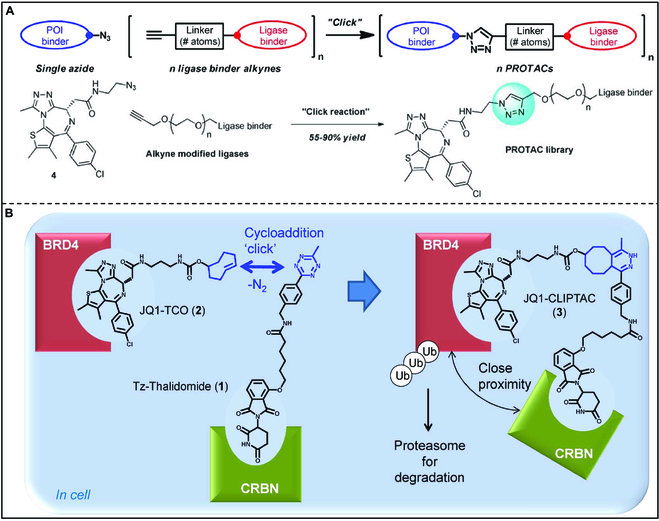
(A) General strategy using “click chemistry” for parallel PROTAC synthesis. Reproduced with permission [163]. Copyright 2018, American Chemical Society. (B) Representative scheme explaining the mode of action of click-formed PROTACs. Reproduced with permission [164]. Copyright 2016, American Chemical Society.

For instance, Amgen Inc. has reported a general strategy for developing PROTAC using bioorthogonal chemistry and constructing a PROTAC molecular library using this strategy (Fig. 21A) [163]. Meanwhile, Astex Pharmaceuticals has reported the successful application of 2 drug precursors to degrade 2 cancer targets, bromine domain protein 4 (BRD4) and ERK1/2, utilizing IEDDA click chemistry to form a hetero-bifunctional molecule in cells (Fig. 21B) [164]. BRD4, a member of the BET family, has been implicated in blood, breast, and colon cancer. ERK1/2 is an extracellular regulatory protein kinase, which is related to nuclear localization and promoting cell proliferation.

**Fig. 22.**
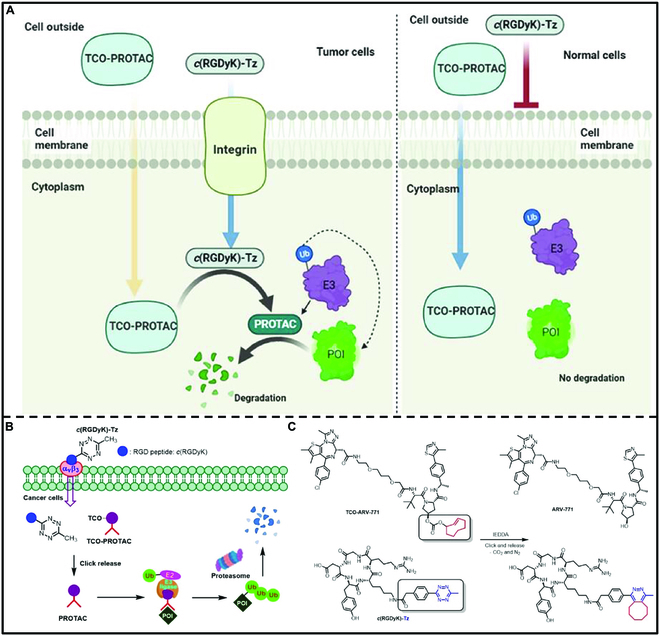
(A) Schematic illustration of bioorthogonal PROTAC prodrugs on-target activation. (B) Bioorthogonal activation and release of TCO-PROTAC prodrugs by αvβ3 integrin-targeted c(RGDyK)-Tz in tumor cells. (C) Structures of TCO-ARV-771 and c(RGDyK)-Tz TCO-Tz and the “click and release” reaction between TCO-ARV-771 and c(RGDyK)-Tz releases the activated ARV-771. Reproduced with permission [167]. Copyright 2016, American Chemical Society.

PROTACs have emerged as a promising therapeutic approach, but there are important questions about the potential toxicity of this approach due to uncontrolled protein degradation and poor ligase-mediated off-target effects [165]. In addition, toxicity due to protein degradation in healthy cells and poor ligase-mediated off-target effects may limit the clinical application of proteins [166]. To address these issues, PROTAC prodrugs are designed to be selectively delivered or activated at the tumor site. Precise control of the degradation activity of PROTACs minimizes potential toxic side effects. Thus, Chang et al. [167] synthesized inactive prodrugs TCO-ARV-771 and TCO-DT2216 by coupling TCO groups to VHL-E3 ubiquitin ligase, and targeted tetrazine (Tz)-modified RGD peptide c(RGDyK)-Tz to integrin αvβ3 biomarkers in cancer cells (Fig. 22). It can be used as an activator of click-to-release PROTAC (*cr*PROTAC), thereby achieving targeted degradation of the POI in cancer cells. The authors verified the click-to-release ability of c(RGDyK)-Tz on TCO-ARV-771 (1.0 mM) and TCO-DT2216 by using UFLC and verified that c(RGDyK)-Tz could selectively deliver corresponding PROTAC to cancer cells through the integrin on the surface of cancer cells by using fluorescence imaging experiments. Western blotting confirmed that c(RGDyK)-Tz can bioorthogonally activate inactive prodrugs to promote BRD4 degradation in HeLa cells through the ubiquitin–proteasome pathway, and the selectivity of BRD4 degradation in other cancer cells with high αvβ3 integrin expression validated the universality of this strategy. The results of the feasibility study of this strategy in this study showed that PROTAC can be selectively activated in a manner dependent on integrin αvβ3 to produce PROTAC to degrade POIs in cancer cells. The *cr*PROTAC strategy is a general-purpose abiotic approach that can selectively induce cancer cell death through the ubiquitin–proteasome pathway.

### Tissue engineering and regenerative medicine

Hydrogels are hydrophilic or amphiphilic 3-dimensional network polymer materials that offer great potential in tissue engineering and regenerative medicine [168]. Their high water content enables them to create a microenvironment akin to the natural extracellular matrix, making them highly valuable [169]. Bioorthogonal reactions, especially catalyst-free click reactions, have emerged as innovative crosslinking strategies for hydrogel development, owing to their rapidity, efficiency, selectivity, and biocompatibility both in vitro and in vivo [170]. Recently, hydrogels and scaffolds based on bioorthogonal reactions have demonstrated their utility in encapsulating cells and bioactive factors, showing great promise in the fields of tissue engineering and regenerative medicine [171,172].

**Fig. 23.**
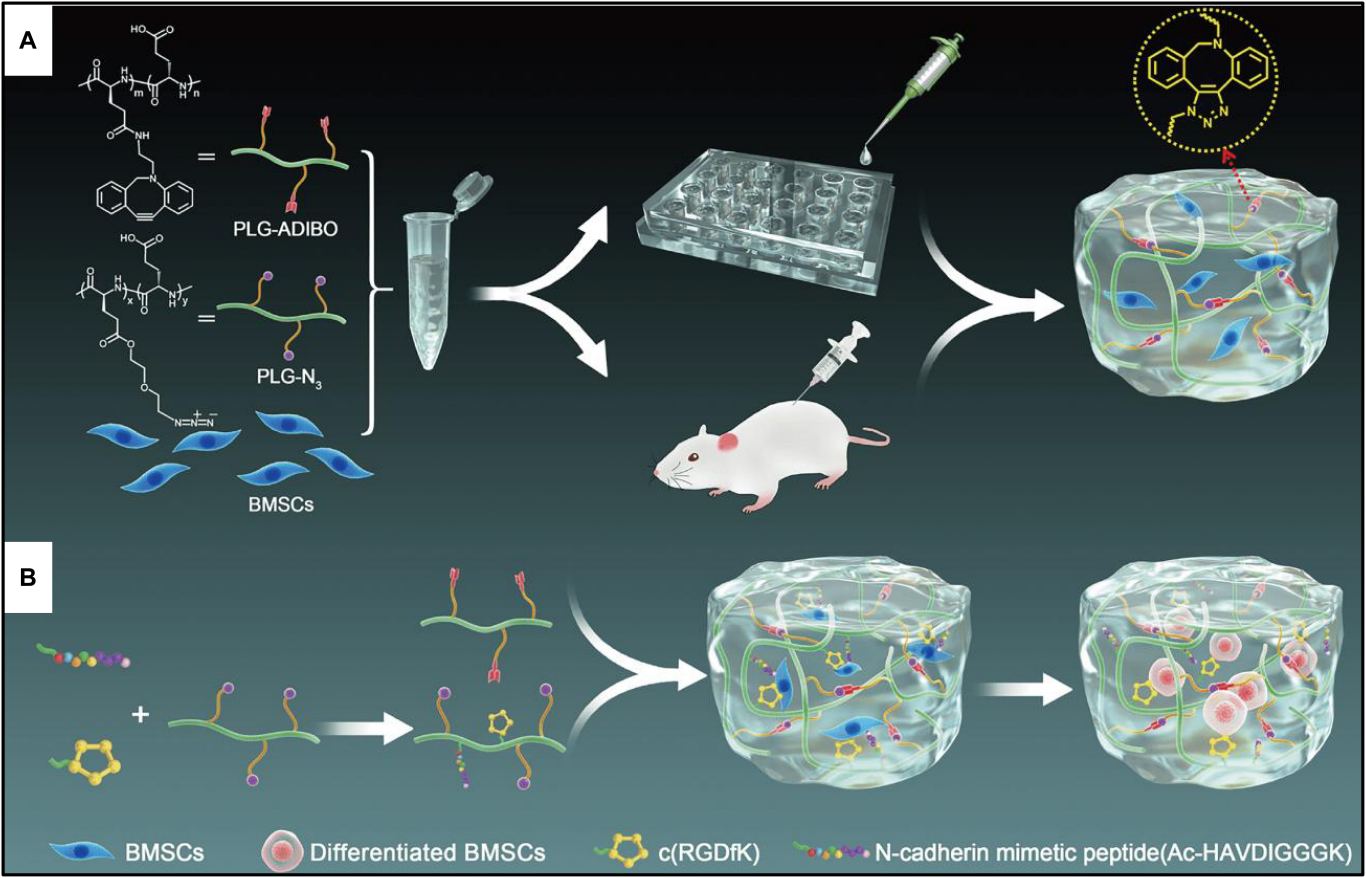
(A) A schematic diagram illustrates the process of forming click-crosslinked polypeptide hydrogels using PLG-N_3_ and PLG-ADIBO, which are intended for use as a cell delivery vehicle. (B) To create bioactive polypeptide hydrogels, bioactive molecules c(RGDfK) and N-Cad are incorporated into the PLG backbone. Reproduced with permission [173]. Copyright 2020, Springer Nature.

Pursuing the objective of crafting bioactive and biodegradable 3D cell culture hydrogel scaffolds, Rong et al. [173] devised a non-catalytic “click” reaction crosslinked poly(L-glutamic acid) (PLG) hydrogel (Fig. 23). This innovative approach entailed the simultaneous incorporation of cell adhesion peptide c(RGDfK) and N-cadherin mimetic peptide (N-Cad) into the hydrogel network, orchestrating dual interactions within the hydrogel-cell system. Employing this bioactive hydrogel as a scaffold material substantially enhanced the adhesion of bone marrow mesenchymal stem cells (BMSCs) to its surface and fostered chondrogenic differentiation of stem cells embedded within the gel. BMSCs demonstrated remarkable survival rates during in vitro culture within the hydrogel. When injected subcutaneously in rats, the hydrogel exhibited complete degradation within 10 weeks and demonstrated excellent histocompatibility. Furthermore, augmenting the PLG side chain with both c(RGDfK) and N-Cad significantly bolstered the hydrogel’s bioactivity. N-cadherin facilitates cell–cell connections and signals exchange via homologous interactions. N-Cad, a synthetic short peptide containing the histidine–alanine–valine domain, has been reported to exhibit binding properties akin to N-cadherin. The study results underscored that covalently linking these 2 peptides to the PLG hydrogel system effectively enhanced BMSC adhesion to the hydrogel’s surface, promoted chondrocyte proliferation within the hydrogel, and, notably, during chondrogenic differentiation of cultured BMSCs, substantially amplified chondrogenic matrix production, as well as the expression of chondrogenic genes and proteins within the hydrogel. In essence, this study introduces an innovative method for constructing a multifunctional 3D cell culture platform.

**Fig. 24.**
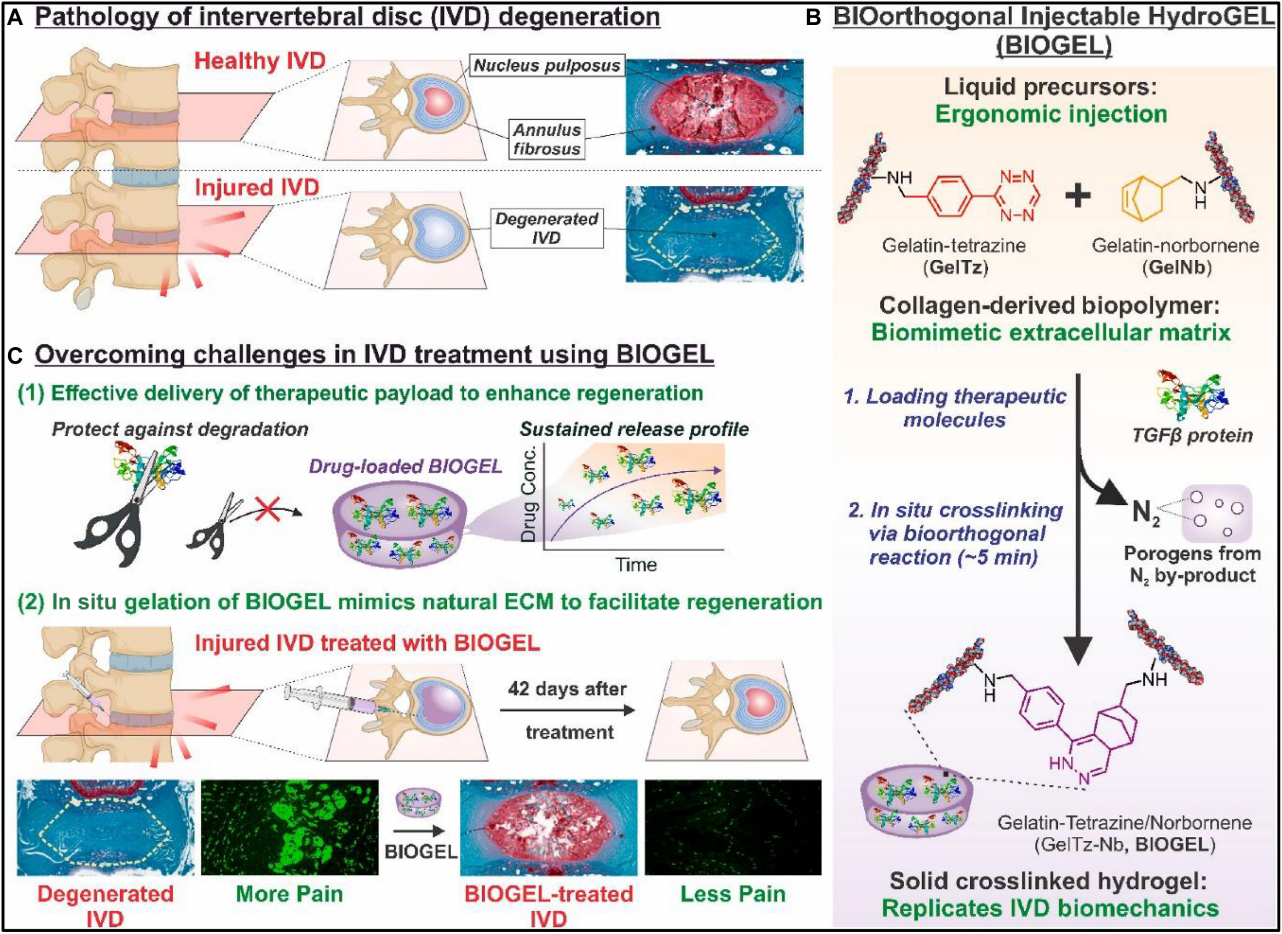
Schematic for injectable BIOGEL for IVD regeneration. (A) IVDs are load-bearing tissues found between 2 vertebrae. (B) Gelatin-tetrazine (GelTz) and gelatin-norbornene (GelNb) are well-suited to form the basis for an injectable BIOGEL. (C) BIOGEL was adapted to treat IVD degeneration as a proof of concept. Reproduced with permission [174]. Copyright 2023, Elsevier.

Luo et al. [174] developed an injectable bioorthogonal hydrogel system, known as BIOGEL, with the primary aim of facilitating tissue regeneration within the intervertebral disc (IVD) (Fig. 24). Specifically, they introduced tetrazine and norbornene functional groups to gelatin, resulting in 2 precursor solutions characterized by their low viscosity across a broad temperature range. These solutions could be easily crosslinked in situ by simple mixing, rendering them suitable for clinical injection scenarios. The BIOGEL also exhibited biomechanical properties akin to those of healthy IVD tissue and displayed a synergistic effect when combined with existing growth factor therapy. Taking TGFβ as an exemplar, it not only achieved a sustained-release effect but also bolstered its anti-inflammatory and regenerative properties, thereby promoting histological and functional recovery in IVD tissue. Due to BIOGEL’s reliance on bioorthogonal chemistry and its use of gelatin from collagen, the system can be readily extended to accommodate other payloads, target various degenerative diseases, and foster tissue regeneration, making it a promising candidate for a wide spectrum of regenerative medicine applications.

**Fig. 25.**
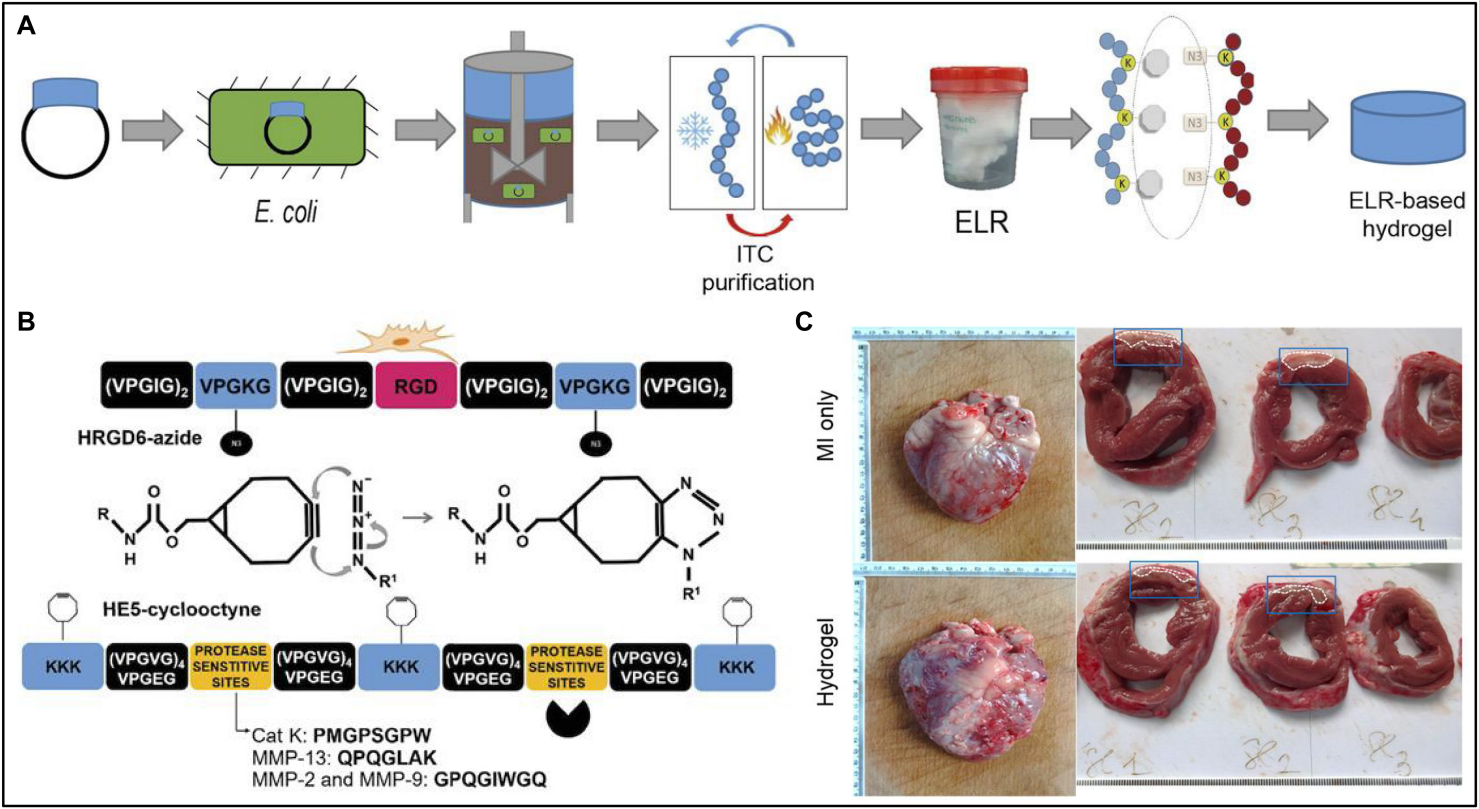
(A) Schematic of ELR recombinant technology synthesis, inverse transition cycling purification, and catalyst-free click chemistry hydrogel formation. (B) Schematic of HR hydrogel formation. (C) Gross heart explant images captured 28 days post-MI depict non-transmural infarcts, denoted by dashed lines enclosed within blue boxes, in both MI-only and hydrogel-treated hearts. Reproduced with permission [175]. Copyright 2021, AAAS.

The Pandit group recently reported the pioneering use of a bioorthogonal reaction-based hydrogel in large animals (Fig. 25) [175]. This hydrogel was applied to a sheep model with non-transmural myocardial infarction (MI). To create this hydrogel, the authors employed 2 elastin-like recombinases (ELRs) crosslinked through copper-free bioorthogonal reaction, both of which were genetically engineered and expressed in *Escherichia coli*. One ELR featured an RGD sequence to facilitate efficient cell adhesion, while the other included a protease-sensitive site susceptible to degradation by enzymes like cathepsin K, MMP-13, MMP-2, and MMP-9, all abundant in the MI site. Additionally, the ELRs were modified with azide and bicyclo[2.1.0]nonyne (BCN) for SPAAC. Gelation occurred within 10 min, and the storage modulus increased at 37 °C, attributed to the inherent characteristics of elastin. Following intramyocardial injection of the hydrogel into the ovine heart afflicted with non-transmural MI, the control group displayed an improved ejection fraction. The hydrogel-treated group exhibited a 44.3% reduction in fibrotic area and a 3.5-fold increase in Nr4a1 expression, an antifibrotic marker within the ischemic core. The authors elucidated that the degradable hydrogel scaffold facilitated endothelial cell sprouting and stromal cell migration, capabilities unattainable with non-degradable hydrogels. Consequently, this led to enhanced angiogenesis and an increased number of capillaries within the MI site, ultimately bolstering the survival of cardiomyocytes post-ischemia. These promising findings with click chemistry-based hydrogels in large animals are paving the path toward future human applications.

## Summary and Perspectives

Bioorthogonal chemistry, known for its outstanding efficiency and accuracy, has broken the boundary between disciplines, bringing the interdisciplinary pace of chemistry, life science, medical pharmacy, and materials science into the functionalism era. The past decades have witnessed the fast development of various bioorthogonal reactions including native chemical ligations, oxime/hydrazone ligations, Staudinger ligations, CuAAC, SPAAC, IEDDA, light-catalyzed bioorthogonal reactions, metal-catalyzed bioorthogonal reactions, etc. These unique reactions have shown great potential in biomedicine like bio-labeling, nucleic acid functionalization, drug discovery, drug activation, synthesis of ADCs, synthesis of PROTACs, etc. The development and application of bioorthogonal reactions have revolutionized our ability to study and manipulate biological systems with high precision and selectivity. These reactions have great potential for advancing our understanding of complex biological processes, as well as for the development of innovative diagnostic tools and targeted therapies in biomedical research and clinical applications. Bioorthogonal chemistry is a springing and innovative field, where scientists have been expanding its connotation and extension for nearly 20 years. From being a complementary reaction to coupling reactions, the bioorthogonal reaction has grown independently and now become the core area of biochemistry, and the potential for development cannot be underestimated.

The bioorthogonal chemical approaches have made synthesis easier and more reliable, thereby contributing to a deeper comprehension of biological mechanisms and the development of more effective and selective treatments. The related work has led to remarkable advances in chemistry, biology, and medicine, making possible work that would otherwise have been impossible. Although the principle of bioorthogonal reaction is simple, it still has room for improvement in several aspects, such as reaction rate, reaction efficiency, substrate accessibility, operation simplicity, and biocompatibility [176]. These factors require enhancement to meet the diverse needs of biological and pharmaceutical basic research as well as clinical research. Besides, the condition is rather harsh on the chemical reactions that qualify. First, the reaction must be carried out at room temperature. Because the reaction is required to take place in a biological environment, where the temperature is usually around room temperature, most of the chemical reactions with high activation energy that require heating are ruled out. Second, the reaction must take place in a liquid environment with a pH near neutral. The most extensive medium in living organisms is water, which cannot be over-acidic or over-alkaline, and this condition also screens out most chemical reactions. Third, the reaction must be sufficiently rapid and efficient. Because chemical reactions occur in living cells or organisms, the concentration of small chemical molecules involved in the reaction cannot be very high, which requires a faster rate and a higher conversion rate. Fourth, it is preferable for the reaction to be conducted without any catalyst, and when necessary, the catalyst should be non-toxic to the biological system or within an acceptable range of toxicity. Last but not least, the reaction is not affected by the various substances in the biological system. This is the hardest part because even a living cell contains a variety of components, some of which have their own highly reactive groups. Despite these limitations, ongoing research and development efforts continue to improve bioorthogonal reactions and overcome these challenges.

The growing array of bioorthogonal reactions presents a challenge in choosing the most suitable ones for specific applications. As previously noted, the bioorthogonal toolbox comprises reactions with distinct strengths and limitations. Therefore, it is crucial for practitioners to prioritize the features most relevant to their specific applications. Under catalytic conditions, CuAAC offers a compact, readily available reaction partner and rapid kinetics. It has been widely employed in in vitro, extracellular, and cell surface applications but is generally ill-suited for intracellular labeling. For intracellular and in vivo scenarios necessitating swift reactivity, tetrazine ligation allows rapid labeling at submicromolar concentrations. It is important to highlight that both reaction partners in tetrazine ligation are relatively bulky, especially with TCO dienophiles, which are also highly reactive. Cycloacetylene [3 + 2] cycloadditions demonstrate efficiency in intracellular settings, enabling the use of azides and other small dipoles as reaction partners. However, their kinetics are generally slower than tetrazine ligation and require higher micromolar to millimolar concentrations. Native chemical ligation serves as the standard for connecting large peptides or protein fragments through native amide bonds, but is limited to in vitro applications. Staudinger linkage offers an alternative approach to forming amide bonds in cellular or in vivo contexts, yet its utility can be hampered by bulky reagents and relatively slow kinetics. Oxime, Pd-catalyzed, and Ru-catalyzed reactions bring novel functional group modifications to bioorthogonal chemical applications. With a few exceptions, these tools are generally best suited for in vitro and extracellular contexts. Light-catalyzed bioorthogonal reaction has emerged as a potent method for achieving precise spatiotemporal control of bioorthogonal reactions in various settings, including live cell applications. Ongoing endeavors to minimize the size of photoinductive groups and shift the activation wavelength toward the red region will broaden the scope of applications. Finally, advancing cleavage reactions to improve reactivity and release kinetics will diversify the toolkit available to researchers.

Nowadays, bioorthogonal chemistry has shown great potential in the fields of imaging, diagnostics, disease treatment, etc. However, more opportunities still exist in the following areas [177]: (a) explore bioorthogonal reactants with better biological stability, or simplify methods by eliminating the need for catalysts (reducing toxicity); (b) applying bioorthogonal reactions to achieve multiple labeling can make it easier to explore biological mechanisms and develop more reliable diagnostics; and (c) improved photoactivated bioorthogonal reactions can minimize damage to the organism and allow deeper imaging inside the organism. In the field of biomedical application, the bioorthogonal reaction is expected to shine in the study of life mechanisms at different levels, from the surface of living cells to the interior of living cells, from a single cell to multiple cells, and from tissues to organisms. In pharmaceutical and other clinical transformation areas, bioorthogonal reaction is expected to be no longer limited to small or large molecules, but also to open new ground in immunotherapy, cell therapy, gene therapy, and other novel therapies in the future. With increasing momentum, bioorthogonal chemistry is empowering research in biology and biomedicine, exerting an important influence on scientists from various disciplines due to its diverse range of applications.
